# Supplementary material for: Computational identification of epifriedelanol and derived analogs from Mikania cordata as potential HMG-CoA reductase inhibitors
Source: PLoS One. 2026 Jan 6;21(1):e0340573. doi: 10.1371/journal.pone.0340573 (PMC12774364; doi:10.1371/journal.pone.0340573)
Supplement: S1 Fig — The cavity ranked first, exhibiting the largest surface area and volume, was selected as the final binding site. A. Cavity ranked 1, B. Cavity ranked 2. (PDF) [file pone.0340573.s001.pdf]

Computational Identification of Epifriedelanol and Derived Analogs from *Mikania cordata* as Potential HMG-CoA Reductase Inhibitors

Supporting information

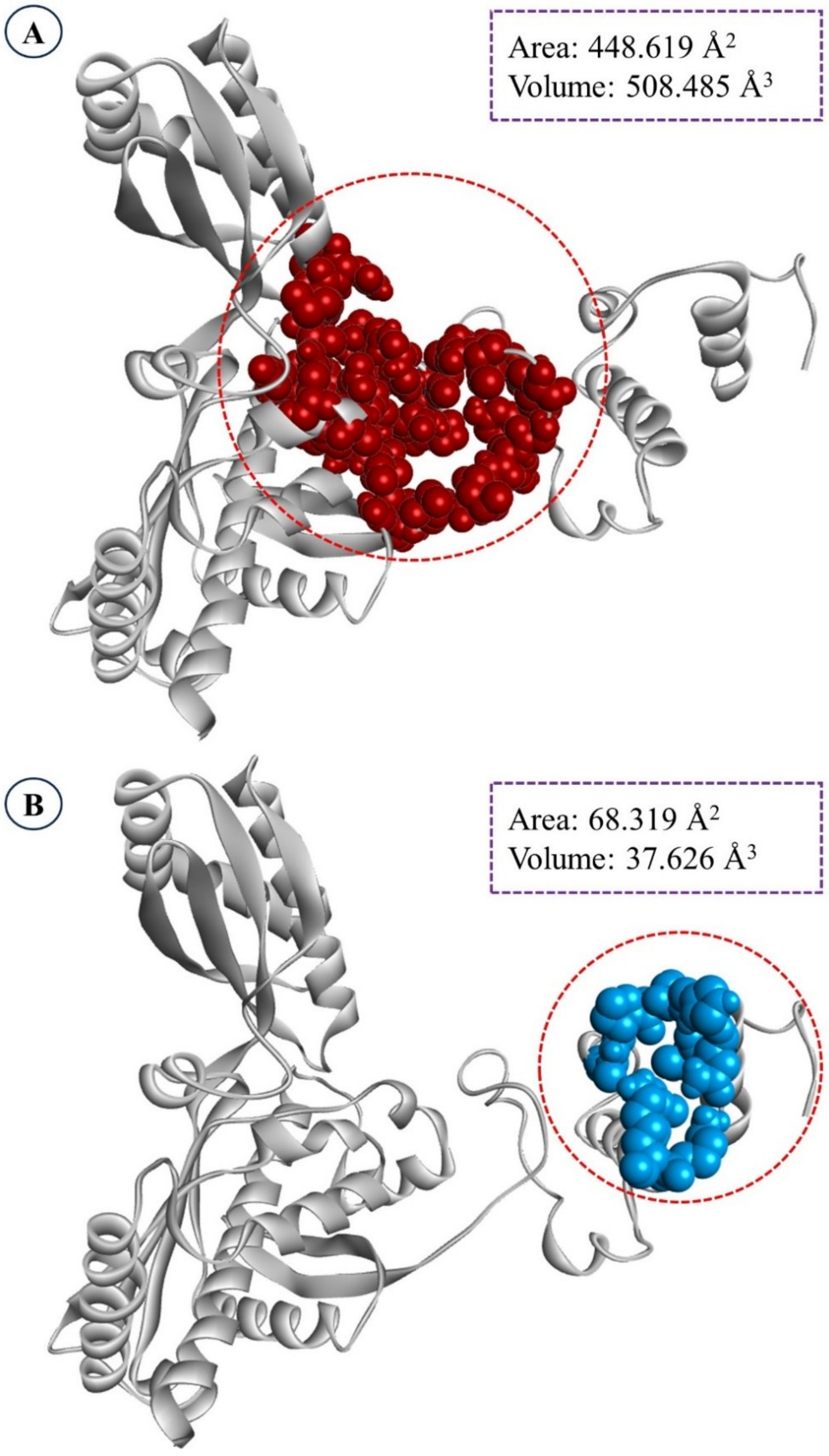

S1 Fig. Identification of the top-ranked active site in the target protein. The cavity ranked first, exhibiting the largest surface area and volume, was selected as the final binding site. A. Cavity ranked 1, B. Cavity ranked 2.
