# Supplementary material for: Computational identification of epifriedelanol and derived analogs from Mikania cordata as potential HMG-CoA reductase inhibitors
Source: PLoS One. 2026 Jan 6;21(1):e0340573. doi: 10.1371/journal.pone.0340573 (PMC12774364; doi:10.1371/journal.pone.0340573)
Supplement: S2 Fig — Numbers are arranged according to the S1 Table. (PDF) [file pone.0340573.s002.pdf]

Computational Identification of Epifriedelanol and Derived Analogs from *Mikania cordata* as Potential HMG-CoA Reductase Inhibitors

Supporting information

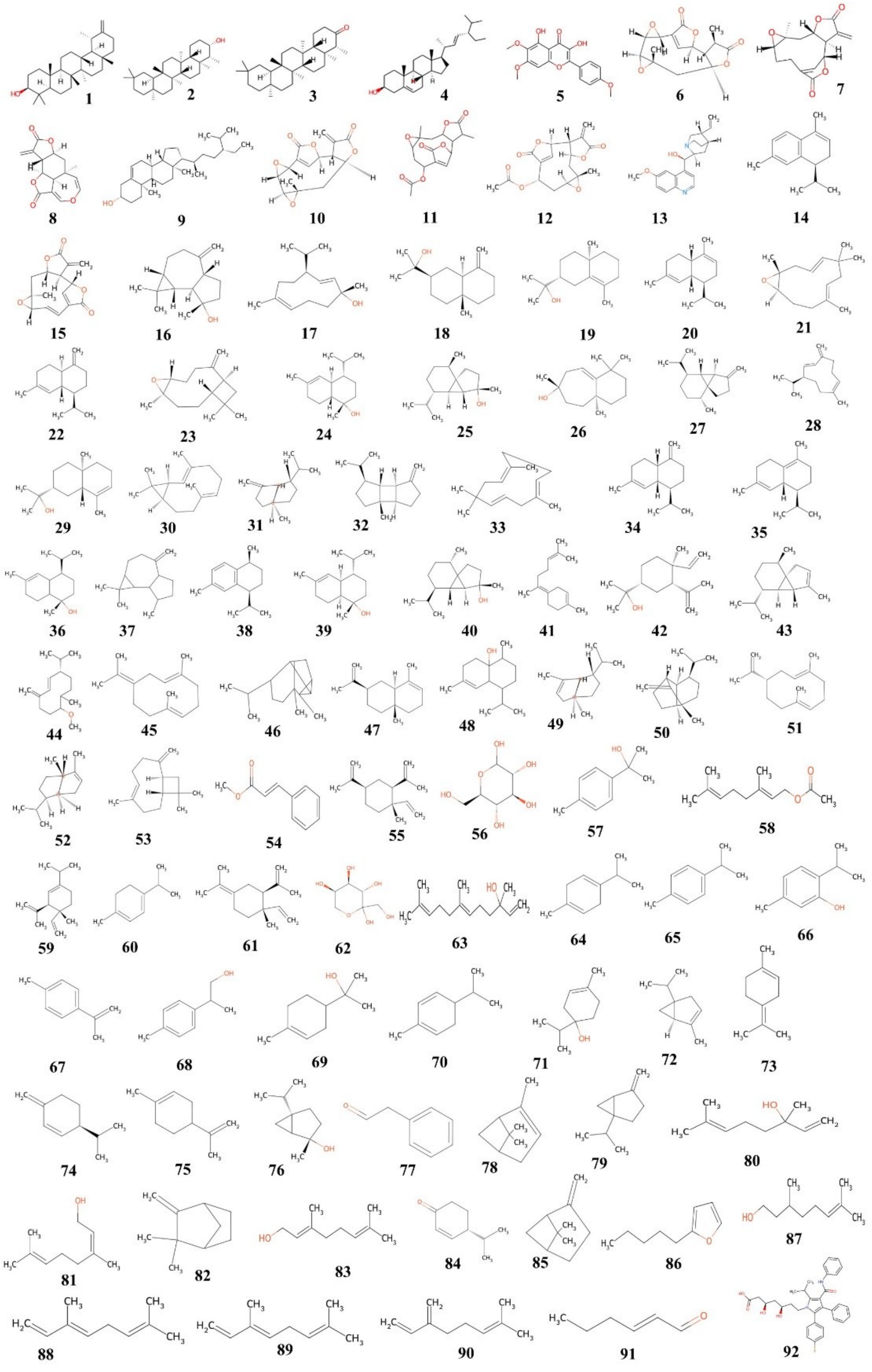

S2 Fig. Two-dimensional chemical structures of the 91 phytocompounds of *M. cordata* and the control drug atorvastatin. Number are arranged according to the Table S1.
