# Supplementary material for: Computational identification of epifriedelanol and derived analogs from Mikania cordata as potential HMG-CoA reductase inhibitors
Source: PLoS One. 2026 Jan 6;21(1):e0340573. doi: 10.1371/journal.pone.0340573 (PMC12774364; doi:10.1371/journal.pone.0340573)
Supplement: S4 Fig — A. EA2, B. EA3, C. Atorvastatin (control). (PDF) [file pone.0340573.s004.pdf]

Computational Identification of Epifriedelanol and Derived Analogs from *Mikania cordata* as Potential HMG-CoA Reductase Inhibitors

Supporting information

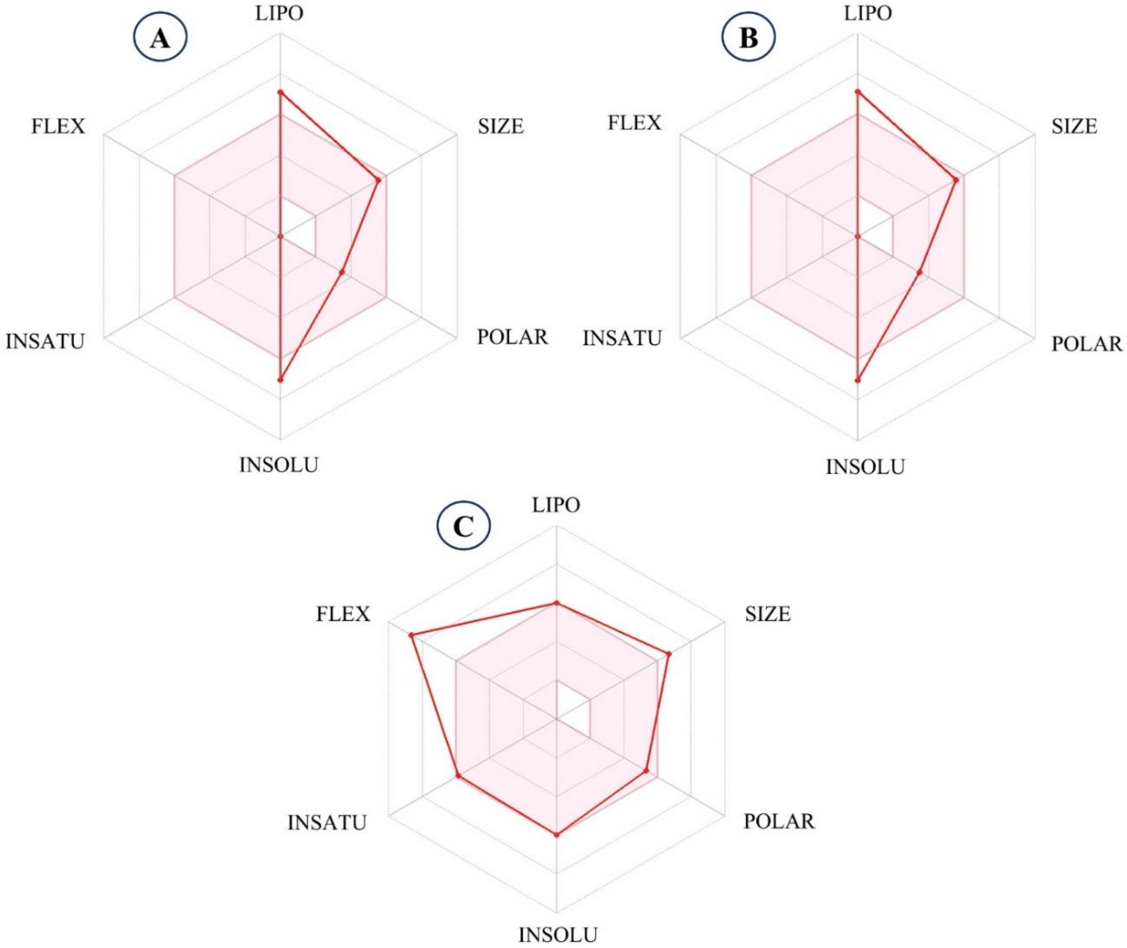

S4 Fig. Bioavailability radar plots showing the key physicochemical properties (lipophilicity, size, polarity, insaturation, solubility, and flexibility) of the epifriedelanol analogs and reference drug. A. EA2, B. EA3, C. Atorvastatin (control).
