# Supplementary material for: Computational identification of epifriedelanol and derived analogs from Mikania cordata as potential HMG-CoA reductase inhibitors
Source: PLoS One. 2026 Jan 6;21(1):e0340573. doi: 10.1371/journal.pone.0340573 (PMC12774364; doi:10.1371/journal.pone.0340573)
Supplement: S6 Fig — A. EA3, B. Atorvastatin (control). (PDF) [file pone.0340573.s006.pdf]

Computational Identification of Epifriedelanol and Derived Analogs from *Mikania cordata* as Potential HMG-CoA Reductase Inhibitors

Supporting information

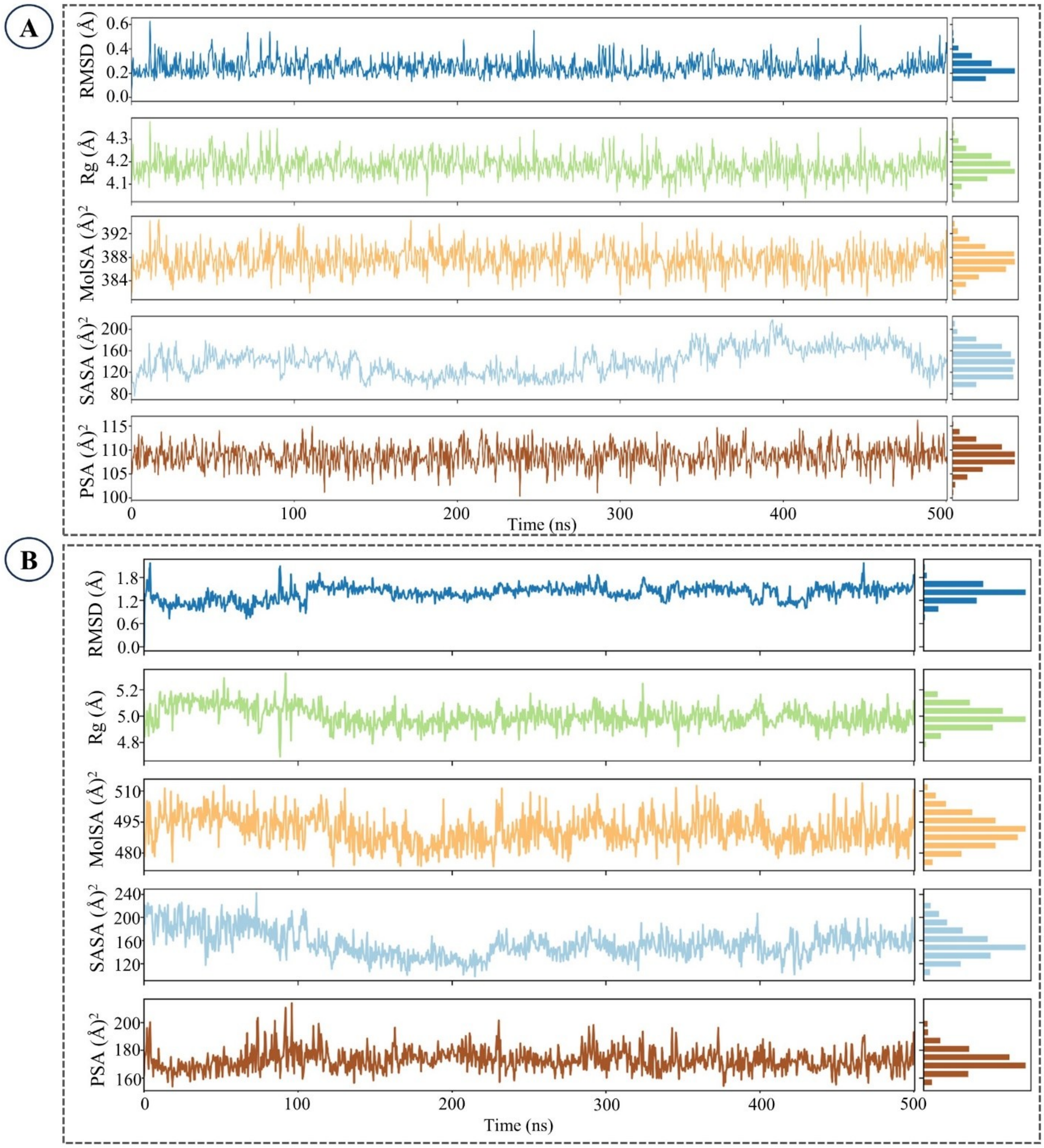

S6 Fig. Evaluation of ligand properties of a lead compound and a control drug. A. EA3, B. Atorvastatin (control).
