# Supplementary material for: Computational identification of epifriedelanol and derived analogs from Mikania cordata as potential HMG-CoA reductase inhibitors
Source: PLoS One. 2026 Jan 6;21(1):e0340573. doi: 10.1371/journal.pone.0340573 (PMC12774364; doi:10.1371/journal.pone.0340573)
Supplement: S1 Table — (PDF) [file pone.0340573.s007.pdf]

Computational Identification of Epifriedelanol and Derived Analogs from *Mikania cordata* as Potential HMG-CoA Reductase Inhibitors

Supporting information

S1 Table. Molecular docking analysis of *M. cordata* phytocompounds and control drug elucidating binding affinity.

Sl. No.	IMPPAT ID/PubChem CID	Name	Chemical formula	Parts used	Molecular weight (g/mol)	Affinity (kcal/mol)
1	IMPHY015081	Taraxasterol	$C_{30}H_{50}O$	Leaf	426.73	−8.8
2	IMPHY011859	Epifriedelanol	$C_{30}H_{52}O$	Root	428.75	−8.6
3	IMPHY011688	Friedelin	$C_{30}H_{50}O$	Root	426.73	−8.1
4	IMPHY014842	Stigmasterol	$C_{29}H_{48}O$	Leaf	412.7	−7.4
5	IMPHY001852	Mikanin	$C_{18}H_{16}O_7$	Stem	344.32	−7.3
6	IMPHY001018	Dihydromikanolide	$C_{15}H_{16}O_6$	Leaf	292.29	−7.0
7	IMPHY002144	Deoxymikanolide	$C_{15}H_{16}O_5$	Leaf	276.29	−7.0
8	IMPHY006921	Miscandenin	$C_{15}H_{14}O_5$	Leaf	274.27	−6.9
9	IMPHY014836	Beta-sitosterol	$C_{29}H_{50}O$	Leaf	414.72	−6.9
10	IMPHY000982	Mikanolide	$C_{15}H_{14}O_6$	Stem	290.27	−6.7
11	IMPHY002835	Dihydroscandenolide	$C_{17}H_{20}O_7$	Leaf	336.34	−6.7
12	IMPHY010420	Scandenolide	$C_{17}H_{18}O_7$	Leaf	334.32	−6.7
13	IMPHY011837	Quinine	$C_{20}H_{24}N_2O_2$	Leaf	324.42	−6.6
14	IMPHY013080	Alpha-calacorene	$C_{15}H_{20}$	Leaf	200.32	−6.5
15	IMPHY007646	Anhydroscandenolide	$C_{15}H_{14}O_5$	Leaf	274.27	−6.4
16	IMPHY007840	Spathulenol	$C_{15}H_{24}O$	Leaf	220.36	−6.3
17	IMPHY011564	Germacra-1(10),5-dien-4-ol	$C_{15}H_{26}O$	Leaf	222.37	−6.3
18	IMPHY011542	Beta-eudesmol	$C_{15}H_{26}O$	Leaf	222.37	−6.2
19	IMPHY011938	Gamma-eudesmol	$C_{15}H_{26}O$	Leaf	222.37	−6.2

Sl. No.	IMPPAT ID/PubChem CID	Name	Chemical formula	Parts used	Molecular weight (g/mol)	Affinity (kcal/mol)
20	IMPHY011659	Alpha-muurolene	$C_{15}H_{24}$	Leaf	204.36	−6.1
21	IMPHY011749	Humulene epoxide II	$C_{15}H_{24}O$	Leaf	220.36	−6.1
22	IMPHY011793	(+)−Gamma-cadinene	$C_{15}H_{24}$	Leaf	204.36	−6.1
23	IMPHY012667	Caryophyllene oxide	$C_{15}H_{24}O$	Leaf	220.36	−6.1
24	IMPHY014906	Cedrelanol	$C_{15}H_{26}O$	Leaf	222.37	−6.1
25	IMPHY003822	Cubebol	$C_{15}H_{26}O$	Leaf	222.37	−6.0
26	IMPHY007367	Widdrol	$C_{15}H_{26}O$	Leaf	222.37	−6.0
27	IMPHY007376	Beta-cubebene	$C_{15}H_{24}$	Leaf	204.36	−6.0
28	IMPHY011586	Germacrane d	$C_{15}H_{24}$	Leaf	204.36	−6.0
29	IMPHY011709	Alpha-eudesmol	$C_{15}H_{26}O$	Leaf	222.37	−6.0
30	IMPHY003616	Bicyclogermacrene	$C_{15}H_{24}$	Leaf	204.36	−5.9
31	IMPHY003719	Beta-copaene	$C_{15}H_{24}$	Leaf	204.36	−5.9
32	IMPHY003977	(−)−Beta-bourbonene	$C_{15}H_{24}$	Leaf	204.36	−5.9
33	IMPHY011761	Humulene	$C_{15}H_{24}$	Leaf	204.36	−5.9
34	IMPHY011792	Gamma-muurolene	$C_{15}H_{24}$	Leaf	204.36	−5.9
35	IMPHY011957	(+)−Delta-cadinene	$C_{15}H_{24}$	Leaf	204.36	−5.9
36	IMPHY012586	(−)−Alpha-cadinol	$C_{15}H_{26}O$	Leaf	222.37	−5.9
37	IMPHY014817	Aromadendrene	$C_{15}H_{24}$	Leaf	204.36	−5.9
38	IMPHY014865	Calamenene	$C_{15}H_{22}$	Leaf	202.34	−5.9
39	IMPHY015128	T-Muurolol	$C_{15}H_{26}O$	Leaf	222.37	−5.9
40	IMPHY003821	1-Epi-Cubebol	$C_{15}H_{26}O$	Leaf	222.37	−5.8
41	IMPHY011839	(Z)−Gamma-bisabolene	$C_{15}H_{24}$	Leaf	204.36	−5.8
42	IMPHY011890	Elemol	$C_{15}H_{26}O$	Leaf	222.37	−5.8

Sl. No.	IMPPAT ID/PubChem CID	Name	Chemical formula	Parts used	Molecular weight (g/mol)	Affinity (kcal/mol)
43	IMPHY014806	Caswell No. 264AB	$C_{15}H_{24}$	Leaf	204.36	−5.8
44	IMPHY016669	Germacrene d, 1,10-epoxide	$C_{16}H_{28}O$	Leaf	236.4	−5.8
45	IMPHY005618	Germacrene B	$C_{15}H_{24}$	Leaf	204.36	−5.7
46	IMPHY009840	Cyclosativene	$C_{15}H_{24}$	Leaf	204.36	−5.7
47	IMPHY011581	Alpha-selinene	$C_{15}H_{24}$	Leaf	204.36	−5.7
48	IMPHY014885	Epicubenol	$C_{15}H_{26}O$	Leaf	222.37	−5.7
49	IMPHY015123	Alpha-copaene	$C_{15}H_{24}$	Leaf	204.36	−5.7
50	IMPHY001941	Sativene	$C_{15}H_{24}$	Leaf	204.36	−5.6
51	IMPHY003695	(−)−Germacrene A	$C_{15}H_{24}$	Leaf	204.36	−5.6
52	IMPHY012168	Alpha-ylangene	$C_{15}H_{24}$	Leaf	204.36	−5.6
53	IMPHY014831	Beta-caryophyllene	$C_{15}H_{24}$	Leaf	204.36	−5.6
54	IMPHY011714	Methyl cinnamate	$C_{10}H_{10}O_2$	Leaf	162.19	−5.5
55	IMPHY010080	Beta-elemene	$C_{15}H_{24}$	Leaf	204.36	−5.4
56	IMPHY014893	D-glucose	$C_6H_{12}O_6$	Root	180.16	−5.4
57	IMPHY002825	2-(4-Methylphenyl)propan-2-ol	$C_{10}H_{14}O$	Leaf	150.22	−5.3
58	IMPHY011647	Geranyl acetate	$C_{12}H_{20}O_2$	Leaf	196.29	−5.3
59	IMPHY013093	Delta-elemene	$C_{15}H_{24}$	Leaf	204.36	−5.3
60	IMPHY011643	Alpha-terpinene	$C_{10}H_{16}$	Leaf	136.24	−5.2
61	IMPHY012921	Gamma-elemene	$C_{15}H_{24}$	Leaf	204.36	−5.2
62	IMPHY014916	D-fructose	$C_6H_{12}O_6$	Root	180.16	−5.2
63	IMPHY015022	Nerolidol	$C_{15}H_{26}O$	Leaf	222.37	−5.2

Sl. No.	IMPPAT ID/PubChem CID	Name	Chemical formula	Parts used	Molecular weight (g/mol)	Affinity (kcal/mol)
64	IMPHY003982	Gamma-terpinene	$C_{10}H_{16}$	Leaf	136.24	−5.1
65	IMPHY006145	P-cymene	$C_{10}H_{14}$	Leaf	134.22	−5.1
66	IMPHY006550	Thymol	$C_{10}H_{14}O$	Leaf	150.22	−5.1
67	IMPHY008150	Alpha-panasinsen	$C_{10}H_{12}$	Leaf	132.21	−5.1
68	IMPHY009866	P-cymen-9-ol	$C_{10}H_{14}O$	Leaf	150.22	−5.1
69	IMPHY012160	Alpha-terpineol	$C_{10}H_{18}O$	Leaf	154.25	−5.1
70	IMPHY014811	Alpha-phellandrene	$C_{10}H_{16}$	Leaf	136.24	−5.1
71	IMPHY011396	4-carvomenthenol	$C_{10}H_{18}O$	Leaf	154.25	−5.0
72	IMPHY011552	Alpha-thujene	$C_{10}H_{16}$	Leaf	136.24	−5.0
73	IMPHY011599	Terpinolene	$C_{10}H_{16}$	Leaf	136.24	−5.0
74	IMPHY011965	(+)−Beta-phellandrene	$C_{10}H_{16}$	Leaf	136.24	−5.0
75	IMPHY014988	Limonene	$C_{10}H_{16}$	Leaf	136.24	−4.9
76	IMPHY016027	Trans-sabinene hydrate	$C_{10}H_{18}O$	Leaf	154.25	−4.9
77	IMPHY007539	Phenylacetaldehyde	C_8H_8O	Leaf	120.15	−4.8
78	IMPHY012061	Alpha-pinene	$C_{10}H_{16}$	Leaf	136.24	−4.8
79	IMPHY012165	Sabinene	$C_{10}H_{16}$	Leaf	136.24	−4.7
80	IMPHY012058	Linalool	$C_{10}H_{18}O$	Leaf	154.25	−4.6
81	IMPHY012654	Nerol	$C_{10}H_{18}O$	Leaf	154.25	−4.6
82	IMPHY014852	Camphene	$C_{10}H_{16}$	Leaf	136.24	−4.6
83	IMPHY014923	Geraniol	$C_{10}H_{18}O$	Leaf	154.25	−4.6

Sl. No.	IMPPAT ID/PubChem CID	Name	Chemical formula	Parts used	Molecular weight (g/mol)	Affinity (kcal/mol)
84	IMPHY003955	4-Isopropenylcyclohex-2-enone, cryptone	$C_9H_{14}O$	Leaf	138.21	−4.5
85	IMPHY012147	Beta-pinene	$C_{10}H_{16}$	Leaf	136.24	−4.5
86	IMPHY005811	2-Pentylfuran	$C_9H_{14}O$	Leaf	138.21	−4.3
87	IMPHY012104	Citronellol	$C_{10}H_{20}O$	Leaf	156.27	−4.3
88	IMPHY012739	(Z)-beta-ocimene	$C_{10}H_{16}$	Leaf	136.24	−4.2
89	IMPHY014835	Beta-e-ocimene	$C_{10}H_{16}$	Leaf	136.24	−4.2
90	IMPHY003485	Myrcene	$C_{10}H_{16}$	Leaf	136.24	−4.0
91	IMPHY011562	2-Hexenal	$C_6H_{10}O$	Leaf	98.14	−3.7
92	**60823 (Control)**	Atorvastatin	$C_{33}H_{35}FN_2O_5$	N/A	558.65	**−7.7**
