# Supplementary material for: Computational identification of epifriedelanol and derived analogs from Mikania cordata as potential HMG-CoA reductase inhibitors
Source: PLoS One. 2026 Jan 6;21(1):e0340573. doi: 10.1371/journal.pone.0340573 (PMC12774364; doi:10.1371/journal.pone.0340573)
Supplement: S2 Table — (PDF) [file pone.0340573.s008.pdf]

Computational Identification of Epifriedelanol and Derived Analogs from *Mikania cordata* as Potential HMG-CoA Reductase Inhibitors

Supporting information

S2 Table. Free binding energy analysis of the top selected phytocompounds of *Mikania cordata*.

**Complexes**	**ΔG Bind (kcal/mol)**	**ΔG Coulomb (kcal/mol)**	**ΔG Covalent (kcal/mol)**	**ΔG Lipo (kcal/mol)**	**ΔG Bind Solv GB (kcal/mol)**	**ΔG Bind vdW (kcal/mol)**
Epifriedelanol-protein complex	−52.3	−3.0	0.1	−20.9	16.6	−44.5
Taraxasterol-protein complex	−48.6	−4.9	0.9	−17.9	17.6	−43.6
Atorvastatin-protein complex	−44.7	3.6	5.1	−27.9	22.9	−46.0
