# Supplementary material for: Computational identification of epifriedelanol and derived analogs from Mikania cordata as potential HMG-CoA reductase inhibitors
Source: PLoS One. 2026 Jan 6;21(1):e0340573. doi: 10.1371/journal.pone.0340573 (PMC12774364; doi:10.1371/journal.pone.0340573)
Supplement: S3 Table — (PDF) [file pone.0340573.s009.pdf]

Computational Identification of Epifriedelanol and Derived Analogs from *Mikania cordata* as Potential HMG-CoA Reductase Inhibitors

Supporting information

S3 Table. Free binding energy calculation of the selected epifriedelanol analogs and control drug.

**Complex**	**ΔG Bind (kcal/mol)**	**ΔG Coulomb (kcal/mol)**	**ΔG Covalent (kcal/mol)**	**ΔG Hbond (kcal/mol)**	**ΔG Lipo (kcal/mol)**	**ΔG Solv GB (kcal/mol)**	**ΔG vdW (kcal/mol)**
EA2-protein complex	−59.8	−13.9	1.4	−1.1	−20.9	22.5	−47.7
EA3-protein complex	−59.7	−13.9	1.4	−1.1	−20.9	22.6	−47.7
Atorvastatin-protein complex	−44.7	3.6	5.1	−0.9	−27.9	22.9	−46.0
