# Supplementary material for: Computational identification of epifriedelanol and derived analogs from Mikania cordata as potential HMG-CoA reductase inhibitors
Source: PLoS One. 2026 Jan 6;21(1):e0340573. doi: 10.1371/journal.pone.0340573 (PMC12774364; doi:10.1371/journal.pone.0340573)
Supplement: Attachment — Submitted filename: Response to reviewers.pdf [file pone.0340573.s011.pdf]

Dr. M. Oliur Rahman
M.Sc. (Dhaka), Ph.D. (Japan), FLS (London)

Professor

Department of Botany
University of Dhaka
Dhaka-1000, Bangladesh
Phone: ++88 01717751718 (Cell); (02) 9661900 (off)
E-mail: oliur.bot@du.ac.bd

Manuscript Number: PONE-D-25-51503

Manuscript Title: "Computational Identification of Epifriedelanol and Derived Analogs from *Mikania cordata* as Potential HMG-CoA Reductase Inhibitors"

Dr. Chandrabose Selvaraj
Academic Editor
PLOS ONE
1265 Battery Street, San Francisco, CA 94111
United States of America

Subject: Response to Reviewers and Submission of Revised Manuscript

Dear Dr. Selvaraj

Warm Greetings.

We would like to extend our sincere gratitude to you and the reviewers for the time and effort dedicated to evaluating our manuscript titled "Computational Identification of Epifriedelanol and Derived Analogs from *Mikania cordata* as Potential HMG-CoA Reductase Inhibitors" (Manuscript ID: **PONE-D-25-51503**). We appreciate your valuable consideration of our work for potential publication in *PLOS ONE.*

The reviewers' insightful comments and constructive feedback have been instrumental in improving our manuscript. We have carefully addressed each point raised, making substantial revisions to enhance clarity, coherence, and scientific rigor. Additionally, grammatical and typographical errors have been corrected, and all changes are highlighted in yellow in the revised manuscript.

Please be informed that the manuscript has been meticulously revised in alignment with the journal's guidelines. Below, we provide our detailed responses to the editorial and reviewers' comments.

RESPONSES TO THE COMMENTS OF THE EDITOR AND REVIEWERS

Editorial Comments

When submitting your revision, we need you to address these additional requirements.

Comment 1. Please ensure that your manuscript meets PLOS ONE's style requirements, including those for file naming. The PLOS ONE style templates can be found at

https://journals.plos.org/plosone/s/file?id=wjVg/PLOSOne_formatting_sample_main_body.pdf and

https://journals.plos.org/plosone/s/file?id=ba62/PLOSOne_formatting_sample_title_authors_affiliations.pdf

Response 1. We have thoroughly revised the manuscript and all associated files to ensure full compliance with PLOS ONE's formatting and style requirements, including file naming conventions, manuscript structure, and consistent referencing throughout the text.

Comment 2. Please note that PLOS One has specific guidelines on code sharing for submissions in which author-generated code underpins the findings in the manuscript. In these cases, we expect all author-generated code to be made available without restrictions upon publication of the work. Please

Dr. M. Oliur Rahman
M.Sc. (Dhaka), Ph.D. (Japan), FLS (London)

Professor

Department of Botany
University of Dhaka
Dhaka-1000, Bangladesh
Phone: ++88 01717751718 (Cell); (02) 9661900 (off)
E-mail: oliur.bot@du.ac.bd

review our guidelines at https://journals.plos.org/plosone/s/materials-and-software-sharing#loc-sharing-code and ensure that your code is shared in a way that follows best practice and facilitates reproducibility and reuse.

Response 2. In accordance with PLOS ONE's guidelines for reproducibility and open science, all author-generated code underlying the findings of this study has been made publicly available in the Zenodo repository and can be accessed via the following DOI: https://doi.org/10.5281/zenodo.17553513

Comment 3. We note that your Data Availability Statement is currently as follows: "All relevant data are available within the manuscript"

Please confirm at this time whether or not your submission contains all raw data required to replicate the results of your study. Authors must share the "minimal data set" for their submission. PLOS defines the minimal data set to consist of the data required to replicate all study findings reported in the article, as well as related metadata and methods (https://journals.plos.org/plosone/s/data-availability#loc-minimal-data-set-definition).

For example, authors should submit the following data:

- The values behind the means, standard deviations and other measures reported;
- The values used to build graphs;
- The points extracted from images for analysis.

Authors do not need to submit their entire data set if only a portion of the data was used in the reported study.

If your submission does not contain these data, please either upload them as Supporting Information files or deposit them to a stable, public repository and provide us with the relevant URLs, DOIs, or accession numbers. For a list of recommended repositories, please see https://journals.plos.org/plosone/s/recommended-repositories.

If there are ethical or legal restrictions on sharing a de-identified data set, please explain them in detail (e.g., data contain potentially sensitive information, data are owned by a third-party organization, etc.) and who has imposed them (e.g., an ethics committee). Please also provide contact information for a data access committee, ethics committee, or other institutional body to which data requests may be sent. If data are owned by a third party, please indicate how others may request data access.

Response 3. All raw data necessary to replicate the results of this study have been made publicly available. These data are provided both as Supporting Information accompanying the revised manuscript and in a stable, open-access repository (Zenodo) at https://doi.org/10.5281/zenodo.17553513

Comment 4. If the reviewer comments include a recommendation to cite specific previously published works, please review and evaluate these publications to determine whether they are relevant and should be cited. There is no requirement to cite these works unless the editor has indicated otherwise.

Response 4. We thank the reviewer for the suggested citations. After careful evaluation, we found the recommended works relevant to our study and have incorporated them appropriately in the revised manuscript.

Dr. M. Oliur Rahman
M.Sc. (Dhaka), Ph.D. (Japan), FLS (London)
Professor

Department of Botany
University of Dhaka
Dhaka-1000, Bangladesh
Phone: ++88 01717751718 (Cell); (02) 9661900 (off)
E-mail: oliur.bot@du.ac.bd

Additional Editor Comments

If reviewers recommend citations that are not relevant to the manuscript, authors are encouraged to decline them. The journal assures that choosing not to include such irrelevant citations will not affect the editorial decision on the manuscript.

Response. Reviewer 2 has recommended two papers for citations. We have found them relevant and cited in the revised manuscript. Thank you.

Reviewer 1

In the submitted manuscript entitled "Computational Identification of Epifriedelanol and Derived Analogs from *Mikania cordata* as Potential HMG-CoA Reductase Inhibitors", the authors virtually screened a total of 92 phytocompounds of this medicinal species targeting the HMG-CoA reductase protein. As well, the authors performed molecular docking computations and molecular dynamics simulations over 500 ns. The manuscript is not well-presented, and the results are not well-discussed. Further technical details regarding the molecular docking calculations must be presented. The performance of the employed docking technique must be evaluated before any calculations based on experimental data. Full technical details regarding molecular docking computations and molecular dynamics simulations should be provided in the revised manuscript. All figures are poorly presented. Consequently, the manuscript in its current form is not suitable for publication in PLOS ONE.

Comment 0: The manuscript must be revised, where some typos and grammatical errors were observed.

Response 0. Thank you for your suggestion. The manuscript has been thoroughly revised, and all typographical and grammatical errors have been corrected.

Comment 1: The introduction provides a general context for the study. However, it does not explain the premises of the work and, more specifically, the rationale of the study. As well, the results should not be positioned in the introduction section.

Response 1. We sincerely thank the reviewer for the insightful suggestion. In the revised manuscript, we have clarified the premises and rationale of the study by explicitly explaining the existing knowledge gap concerning the absence of systematic investigations on *M. cordata* phytoconstituents as HMG-CoA reductase inhibitors. The Introduction now emphasizes the scientific reasoning for selecting *M. cordata*, highlighting its well-documented traditional medicinal uses, broad-spectrum bioactivities, and pharmacological relevance to cardiovascular health.

Additionally, we have carefully rephrased all statements in the Introduction to focus on study objectives rather than outcomes, thereby avoiding any implication of results being reported in this section. These revisions ensure that the Introduction now clearly presents the rationale, objective, and novelty of the study in alignment with the journal's guidelines.

Comment 2: Could the authors clarify the rationale for selecting this older PDB entry, despite the availability of more recent structures with improved resolution?

Response 2. We thank the reviewer for this insightful comment. The PDB structure **1HWK** (Istvan and Deisenhofer, 2001) was selected because it represents the first and most extensively validated X-ray crystal structure of human HMG-CoA reductase complexed with a statin inhibitor. With a resolution of 2.22 Å, 1HWK has served as a benchmark model in numerous molecular docking, inhibitor design, and molecular dynamics studies over the past two decades (Ali et al., 2025; Bano

et al., 2025; Suhail et al., 2025). Its longstanding and consistent use in literature supports its structural reliability and reproducibility for computational analyses.

We are aware that more recent structures, such as **8PKN** (released January 2025; Cryo-EM, 2.26 Å) and **8S6B** (released February 2025; Cryo-EM, 2.06 Å) (Karuppasamy and van Rooyen, 2025). However, both models were determined by single-particle Cryo-EM, exhibit **shorter sequence coverage (**423 amino acids**)**, and represent different aggregation states (particle-based reconstructions). In contrast, 1HWK provides a complete 467-residue catalytic domain obtained via high-quality X-ray diffraction, with well-defined refinement statistics (R-Value Work = 0.212, R-Value Free = 0.235).

Therefore, despite the recent availability of Cryo-EM structures with comparable or slightly improved nominal resolution, 1HWK remains the most comprehensive, experimentally validated, and widely cited crystal structure of human HMG-CoA reductase-statin complex. This makes it the most appropriate and consistent choice for structure-based molecular docking and inhibitor interaction analyses in the present study. The rationale for this selection has been discussed in the Discussion section and is highlighted in yellow in the revised manuscript.

Ali, S., Ali, U., & Jan, M. I. (2025). Hypolipidemic and HMG-CoA reductase inhibitory effects of 15-oxoursolic acid from *Rhododendron arboreum*: An *in-vivo* and *in-silico* study. *Drug Development and Industrial Pharmacy*, 1-12.

Bano, S., Ansari, J. A., Ahsan, F., & Khan, A. R. (2025). Molecular docking, phytochemical screening and evaluation of *Balliospermum montanum* roots for its antihyperlipidemic potential. *Cell Biochemistry and Biophysics*, 1-18.

Istvan, E. S., & Deisenhofer, J. (2001). Structural mechanism for statin inhibition of HMG-CoA reductase. *Science*, *292*(5519), 1160-1164.

Karuppasamy, M., & van Rooyen, J. (2025). Cryo-EM structures of apo and atorvastatin-bound human 3-hydroxy-3-methylglutaryl-coenzyme A reductase. *Structural Biology and Crystallization Communications*, *81*(3), 118-122.

Suhail, P., Joy, J., Musliyarakath, N., Padmaraj, C. P., Theruvath, A., & Varghese, N. (2025). Bioactive compounds from *Carica papaya* (Papaya) fruit peel target key enzymes in diabetes and dyslipidemia: A molecular insight. *Biomedical and Biotechnology Research Journal*, *9*(3), 246-254.

Comment 3: Why did the authors utilize CASTp for identifying the binding site, although the binding site of this protein is already known?

Response 3. We sincerely thank the reviewer for this valuable comment. Although the catalytic site of HMG-CoA reductase complexed with statins (e.g., Atorvastatin) is well-established, several recent studies have identified additional druggable pockets and regulatory interfaces within this enzyme (PDB ID 1HWK). Therefore, in the present study, we employed the CASTp server to identify all possible surface-accessible cavities and to evaluate the likelihood of alternative or allosteric binding regions for the selected natural compounds.

Our CASTp-based prediction revealed a prominent cavity encompassing residues such as Tyr517, Ile531, Met534, and Pro813, which partially overlaps with residues reported as critical for dimer formation and stability by Gesto et al. (2014), including Ile531 and Met534. Furthermore, Kanu et al. (2024) demonstrated that natural green tea polyphenols (EGCG, ECG, EC) bind to Glu528, Met534, Ile536, and Lys735, distinct from the canonical Atorvastatin site, yet exhibit strong inhibitory potential against HMG-CoA reductase.

In light of these findings, the CASTp-derived pocket was considered a biologically meaningful and experimentally supported region for ligand docking, enabling the exploration of potential alternative inhibition mechanisms of the enzyme beyond the classical catalytic site. Notably, our results showed that both the control (Atorvastatin) and the phytochemicals bound within the same CASTp-

predicted cavity, reinforcing the reliability of our docking setup. We have discussed the reason of using CASTp for identifying the binding site, highlighted in yellow, in the Discussion section.

Gesto, D. S., Cerqueira, N. M. F. S. A., Ramos, M. J., & Fernandes, P. A. (2014). Discovery of new druggable sites in the anti-cholesterol target HMG-CoA reductase by computational alanine scanning mutagenesis. *Journal of Molecular Modeling*, *20*(4), 2178.

Kanu, V. R., Pulakuntla, S., Kuruvalli, G., Aramgam, S. L., Marthadu, S. B., Pannuru, P., ... & Vaddi, D. R. (2024). Anti-atherogenic role of green tea (*Camellia sinensis*) in South Indian smokers. *Journal of Ethnopharmacology*, *332*, 118298.

Comment 4: What is the exhaustiveness number utilized for performing molecular docking computations?

Response 4. We thank the reviewer for this query. The exhaustiveness parameter in EasyDock Vina v.2.237 was set to 9, which is the fixed default value in the software. This value ensures sufficient sampling of the ligand conformational space during docking. We have explicitly added this detail explicitly in the subsection "*2.4. Molecular docking*" of the revised manuscript.

Comment 5: The authors claimed that "the ADMET evaluation was conducted using SwissADME to assess the drug-likeness properties of the selected compounds". However, there is a big difference between ADMET and drug likeness properties. Please revise and correct.

Response 5. Thank you very much for your critical observation. We fully agree that ADMET and drug-likeness represent distinct aspects of compound evaluation. Accordingly, we have revised the Methodology section to clarify this distinction. The updated text now reads:

> "The pharmacokinetic and drug-likeness properties of the selected compounds were evaluated using the SwissADME server [45]. Subsequently, toxicity parameters were predicted separately using the STopTox server to assess potential adverse effects, completing the comprehensive ADMET evaluation [46]."

This revision ensures that the methodological description accurately reflects the separate evaluation of pharmacokinetic, drug-likeness, and toxicity properties.

Comment 6: Full technical details regarding molecular dynamics simulations should be provided in the revised manuscript.

Response 6. Thank you for the suggestion. In the revised manuscript, the MD simulation methodology [subsection *2.8 Molecular Dynamics (MD) Simulation*] has been substantially expanded to include complete technical details, such as ensemble conditions, thermostat and barostat controls, long-range electrostatics treatment, sampling frequency, and post-MD analytical procedures. The revised methodology part now comprehensively describes the simulation setup, ensuring transparency and reproducibility.

Comment 7: How did the authors compute the atomic charges of the investigated compounds prior to molecular dynamics simulations?

Response 7. We thank the reviewer for raising this important point. As described in the subsection 2.8, partial atomic charges for all ligands were automatically assigned during system preparation in Maestro, ensuring full compatibility with the OPLS4 force-field parameters used in Desmond. This procedure ensures physically consistent charge representation throughout the simulation workflow.

Comment 8: Table 1 is very long. It should be summarized, and other data transported into supporting information.

Dr. M. Oliur Rahman
M.Sc. (Dhaka), Ph.D. (Japan), FLS (London)

Professor

Department of Botany
University of Dhaka
Dhaka-1000, Bangladesh
Phone: ++88 01717751718 (Cell); (02) 9661900 (off)
E-mail: oliur.bot@du.ac.bd

Response 8. Thank you for your valuable suggestion. We have shortened Table 1 by retaining only the top 24 phytocompounds considering the binding affinely of over -6.0 kcal/mol, while the complete dataset comprising all 91 phytocompounds along with the control drug Atorvastatin has been moved to the supplementary section (Table S1). Additionally, following the recommendation of Reviewer 2, two new columns (Compound name and Plant part used) have been incorporated into both Table 1 and Table S1.

Comment 9: Molecular interactions of the predicted docking poses of the identified analogs should be detailed in the revised manuscript.

Response 9. Thank you for your constructive suggestion. In response, the molecular interaction profiles of the identified analogs have been elaborated in detail in the revised manuscript (*Section 3.5.2. Molecular interaction analysis of structural analogs of Epifriedelanol*). The updated section now provides a comprehensive description of the docking poses of EA2 and EA3, including the specific binding site residues, interaction types (pi–alkyl and carbon–hydrogen interactions), and corresponding bond distances.

Comment 10: What is the benefit of computing binding energy after docking calculations?

Response 10. We thank the reviewer for the comment regarding the benefit of computing binding energy after docking. Post-docking MM/GBSA calculations provide a quantitative estimate of ligand-protein binding affinity, enabling efficient prioritization of potential leads. In our study, this approach allowed us to identify Epifriedelanol as the most promising phytocompound (having the best free binding energy) from *Mikania cordata* ($\Delta G\_bind = -52.3$ kcal/mol), while other candidates such as Taraxasterol (having inferior free binding energy than Epifriedelanol) were eliminated despite favorable ADMET profiles (Table S2). Structural analogs EA2 and EA3 were then shortlisted based on their superior binding energies (−59.8 and −59.7 kcal/mol) relative to the control drug Atorvastatin (−44.7 kcal/mol). This workflow is consistent with previous studies; for example, Shimu et al. (2022) employed MM/GBSA immediately after docking to select promising phytochemical inhibitors of Dengue NS2B/NS3 protein, and Ahmed et al. (2022) used a similar approach to prioritize natural compounds from *Piper betle* against alpha-amylase and alpha-glucosidase prior to molecular dynamics simulations. These examples demonstrate that post-docking MM/GBSA calculations are a widely accepted and effective strategy for lead compound prioritization before performing computationally intensive MD simulations.

Ahmed, S., Ali, M. C., Ruma, R. A., Mahmud, S., Paul, G. K., Saleh, M. A., ... & Islam, M. R. (2022). Molecular docking and dynamics simulation of natural compounds from betel leaves (*Piper betle* L.) for investigating the potential inhibition of alpha-amylase and alpha-glucosidase of type 2 diabetes. *Molecules*, *27*(14), 4526.
Shimu, M. S. S., Mahmud, S., Tallei, T. E., Sami, S. A., Adam, A. A., Acharjee, U. K., ... & Kim, B. (2022). Phytochemical compound screening to identify novel small molecules against dengue virus: A docking and dynamics study. *Molecules*, *27*(3), 653.

Comment 11: The number of tables is very huge. The non-informative tables should be transported into the supporting information.

Response 11. Thank you for your thoughtful suggestion. We have moved Table 4 and Table 8 to the Supplementary Information section, where they are now presented as Table S2 and Table S3, respectively. The remaining tables have been retained in the main manuscript, as they contain essential and informative data supporting the study's main findings.

Comment 12: Post-MD calculations should include binding energy per frame, center-of-mass, and hydrogen bond analyses..., etc.

Dr. M. Oliur Rahman
M.Sc. (Dhaka), Ph.D. (Japan), FLS (London)
Professor

Department of Botany
University of Dhaka
Dhaka-1000, Bangladesh
Phone: ++88 01717751718 (Cell); (02) 9661900 (off)
E-mail: oliur.bot@du.ac.bd

Response 12. Thank you for your valuable recommendations. In the revised manuscript, we have incorporated per-frame binding energy, center-of-mass (COM), and hydrogen bond analyses as suggested. Additionally, ligand strain energy calculations have been included to provide a more comprehensive post-MD evaluation. The corresponding results are presented in the newly added Figures 8 and 9 and Table 7, which have been appropriately cited in the main text.

Comment 13: The binding energy calculations over MD simulations should be computed using the MM-GBSA or MM-PBSA approach in the revised manuscript.

Response 13. We appreciate the reviewer's valuable suggestion. In the revised manuscript, binding free energy calculations were performed following molecular dynamics (MD) simulations using the MM/GBSA approach. A new subsection has been added in the Methodology (*Section 2.9, "Post-MD MM/GBSA analysis*") and the corresponding results are presented in Section 3.7 ("Post-MD free binding energy evaluation"). Additionally, a new figure (Fig. 9) has been included to illustrate the per-frame variation of binding free energy across the simulation trajectory. The detailed energetic components of the MM/GBSA calculations ($\Delta G\_Coulomb$, $\Delta G\_Covalent$, ΔG_vdW, etc.) are summarized in *Table 7 (newly added*).

Comment 14: The performance of the employed docking technique must be evaluated before any calculations based on experimental data.

Response 14. We sincerely thank the reviewer for this valuable comment. To evaluate the performance of the employed docking protocol, a known inhibitor-based validation was performed using Atorvastatin, the reference ligand of HMG-CoA reductase. As the present study focused on the CASTp-predicted cavity (the reason behind selecting CASTp pocket has been explained in the response 3), which differs from the canonical co-crystallized binding site, Atorvastatin was re-docked into the CASTp-defined pocket using the same docking parameters as applied for the phytochemical screening.

The redocked pose was then aligned with the original pre-docking conformation (PubChem pose) using PyMOL, and the root-mean-square deviation (RMSD) was calculated. The redocked conformation closely reproduced the original geometry of the ligand with an **RMSD of 2.163 Å** (*Figure 1, newly added*), confirming the reliability and reproducibility of the employed docking setup for the CASTp-defined pocket. This known inhibitor validation demonstrates that the docking parameters and grid settings were appropriately optimized for accurate pose prediction within the selected cavity.

Comment 15: The number of figures is very large in the current manuscript; non-informative figures should be transported into the supporting information.

Response 15. Thank you for your suggestion. In the revised manuscript, as per suggestion, the previous Figures 1, 4, 7, 12, and 13 have been transferred to the Supporting Information section as Figures S1, S3, S4, S5, and S6, respectively. Additionally, following the reviewer's recommendation for enhanced validation analyses including docking protocol validation, per-frame binding energy (MM/GBSA), hydrogen bond, and center-of-mass (COM) analyses, **three new figures** (**Figures 1, 8,** and **9**) have been incorporated. As a result, the total number of figures has been streamlined from previous 16 to present 11 in the revised manuscript.

Comment 16: RMSF estimates the apo-receptor and ligand-soaked complex variance throughout the simulation time. However, the data are presented in Figure 13.

Response 16. We would like to clarify that the RMSF analysis in our study was performed specifically on the ligand-bound protein complexes to evaluate the flexibility of key binding site residues upon ligand binding. The apo-protein system was not included, as our primary objective was to compare residue-level fluctuations among different ligand-bound complexes rather than between apo and holo forms. The RMSF data are correctly presented in Figure 6E of the revised manuscript, which illustrates the residue flexibility profiles of the Epifriedelanol-, EA2-, EA3-, and Atorvastatin–protein complexes. The previous Figure 13 mentioned in the above comment represents ligand property evaluations and is unrelated to RMSF analysis. We have carefully verified and confirmed that all figure numbers and citations are accurate in the revised version.

Comment 17: According to the post-MD analyses, the investigated compounds are not stable in the binding site of the investigated protein.

Response 17. We appreciate the reviewer's careful scrutiny of the Molecular Dynamics (MD) results and acknowledge that the global protein Cα RMSD (specifically the mean of 9.59 Å for EA2) could initially suggest system instability. We concur that reliance on Cα RMSD alone can be misleading, as it captures both local protein conformational changes and potential ligand dissociation. To provide a robust assessment, we have integrated six orthogonal, ligand-centric, and structural compactness analyses over the **500 ns** trajectories. These metrics support the conclusion that all ligands, including EA2, maintained stable, compact, and persistent binding within the active site, exhibiting **local conformational adaptation rather than dissociation**.

The stability of the protein-ligand complexes is strongly supported by the consistency across six independent analyses, demonstrating that the lead compounds often perform better than the control drug, atorvastatin, in key metrics of binding stability and compactness.

A. Ligand-Centric Positional Stability (Binding Retention)

Ligand-relative RMSD: All test compounds showed lower mean fluctuations compared to the control, Atorvastatin (6.55 ± 0.66 Å). Specifically, EA2 (4.05 ± 0.82 Å) demonstrated stable conformation and movement acceptable for a flexible ligand within the active site (Please refer to the **Figures S5 and S6** in the supplementary file).

Protein-Ligand Center-of-Mass (PL-COM) Distance: This metric directly refutes major displacement. Among all compounds, EA2 exhibited the lowest mean distance (1.63 ± 0.44 Å) with the least fluctuation. This suggests a highly consistent and close binding orientation throughout the 500 ns trajectory, contrasting with Atorvastatin, which showed the highest mean distance (2.05 ± 0.36 Å), indicating greater flexibility within the pocket (Please refer to the newly added Figure 8E).

B. Structural Compactness and Solvent Shielding

Radius of Gyration (Rg): All test complexes maintained consistent and stable structural compactness. The Rg values for the lead compounds (Epifriedelanol: 4.29 ± 0.03 Å; EA2: 4.21 ± 0.05 Å; and EA3: 4.18 ± 0.05 Å) were all lower than that of Atorvastatin (4.99 ± 0.08 Å), indicating a more compact overall structure upon binding (Please refer to the Figures S5 and S6).

Molecular Surface Area (MolSA): This metric remained steady with low standard deviations, directly confirming stable molecular surface exposure. The mean MolSA for EA2 (388.18 ± 2.19 Å^2) and the other lead compounds was considerably lower than Atorvastatin (490.85 ± 7.46 Å^2), suggesting the formation of more compact complexes with less exposed surface area (Please refer to the Figures S5 and S6).

Solvent Accessible Surface Area (SASA): Lower SASA values signify reduced solvent exposure, confirming the ligands are stably buried. The SASA for EA2 (124.38 ± 25.81 Å^2) was lower than the control (154.33 ± 24.26 Å^2), supporting a reduced solvent exposure and compact binding conformation (Please refer to the Figures S5 and S6).

C. Interaction Persistence

Hydrogen-Bond Cumulative Count: While Atorvastatin showed the highest cumulative count (1425), EA2 (896) and EA3 (940) demonstrated a significant and persistent number of polar contacts throughout the simulation, supporting continued engagement with the active site residues (Please refer to the **newly added Figures 8A–D**).

In summary, the increased global Cα RMSD for EA2 is interpreted as local conformational adaptation and protein flexibility, which is a normal aspect of dynamic binding. The consistency and low fluctuations observed across six independent, ligand-centric metrics (PL-COM distance, Ligand-relative RMSD, Rg, MolSA, SASA, and H-bonds) provide strong quantitative evidence that all the lead compounds maintained stable, compact, and persistently bound conformations throughout the simulation. We have revised the manuscript to reflect this detailed interpretation. The Molecular Dynamics (MD) simulation section of the results part have been thoroughly revised, highlighted in yellow with additional figure (**Figure 8**). We have further discussed this observation in the discussion section, highlighted in yellow.

Comment 18: The quality of the presented figures must be improved.

Response 18. Thank you for your suggestion. We have revised all the previous figures, improved quality, and ensured consistency and readability. The figures in the PDF built by the submission system seems blurry. But the original figures are not blurry, we have submitted 300 DPI high-resolution TIF files for all the figures following PACE (Preflight Analysis and Conversion Engine) as per guidelines of PLOS ONE and the supplementary figures as well. In the PDF built by the submission system, there is a hyperlink in the top right corner of the figure containing page, saying "Click here to access/download;Figure;Fig1.tif", please click on the link (as shown in the submission system-generated PDF) to download the high-resolution figure. As the figures have high-resolution and bigger size, the submission system might reduce the resolution in the PDF, to keep the PDF in smaller size.

Comment 19: Negative sign must be replaced by a minus sign in all manuscripts.

Response 19. In the revised manuscript, all negative signs have been replaced with proper minus signs (−) throughout the text, tables, and figures to ensure typographical accuracy and consistency. Thank you.

Comment 20: Reference style must be revised. There are versatile omitted details.

Response 20. Thank you for your suggestion. In the revised manuscript, all references have been thoroughly reviewed and corrected to ensure full compliance with the journal's reference style and formatting requirements. Missing details have been added, and all citations have been standardized to maintain consistency throughout the manuscript.

Comment 21: The conclusion section must be rewritten to be more informative and unveil the most beneficial outcomes.

Response 21. Thank you for your comment. We have rewritten the conclusion to make it more informative and to highlight the most beneficial outcomes of the study.

Dr. M. Oliur Rahman
M.Sc. (Dhaka), Ph.D. (Japan), FLS (London)
Professor

Department of Botany
University of Dhaka
Dhaka-1000, Bangladesh
Phone: ++88 01717751718 (Cell); (02) 9661900 (off)
E-mail: oliur.bot@du.ac.bd

Comment 22: All docking scores and binding energies should be in one decimal unit.

Response 22. Thank you. All the docking scores and binding energies have been kept in one decimal unit.

Comment 23: The number of keywords should be decreased to at most five.

Response 23. We have reduced the number of keywords and kept five keywords (maximum).

Comment 24: Did the authors investigate the protonation state of the titrable amino acids before docking?

Response 24. We thank the reviewer for highlighting the importance of receptor protonation states. Prior to docking, the receptor structure (PDB ID: 1HWK) was prepared using AutoDockTools v1.5.6, where **polar hydrogens were added and Kollman partial charges were assigned**. The protein structure was subsequently energy-minimized using the **GROMOS96 43B1 force field in SWISS-PDB Viewer** to relieve steric clashes, optimize side-chain geometry, and achieve a low-energy conformation. While **explicit pKa calculations were not performed**, this preparation approach is consistent with widely adopted protocols in recent studies of protein-ligand docking (Gonfa et al., 2023; Mahmud et al., 2021; Azmal et al., 2024). We have revised the subsection "2.1. Protein preparation" with additional details and references.

Azmal, M., Paul, J. K., Prima, F. S., Talukder, O. F., & Ghosh, A. (2024). An in silico molecular docking and simulation study to identify potential anticancer phytochemicals targeting the RAS signaling pathway. *PLoS One*, *19*(9), e0310637.

Gonfa, T., Temesgen, A., Erba, O., Mengesha, E. T., & Sivasubramanian, M. (2023). Phytochemicals analysis, in vitro antibacterial activities of extracts, and molecular docking studies of the isolated compounds from *Melhania zavattarii* Cufod leaves. *Journal of Tropical Medicine*, *2023*(1), 8820543.

Mahmud, S., Mita, M. A., Biswas, S., Paul, G. K., Promi, M. M., Afrose, S., ... & Saleh, A. (2021). Molecular docking and dynamics study to explore phytochemical ligand molecules against the main protease of SARS-CoV-2 from extensive phytochemical datasets. *Expert Review of Clinical Pharmacology*, *14*(10), 1305-1315.

Comment 25: Did the authors investigate the protonation state of the ligands before docking computations?

Response 25. We thank the reviewer for highlighting the importance of ligand protonation states. Prior to docking, all ligands were **energy-minimized using OpenBabel v3.1.1.1 with the MMFF94 force field and the steepest descent algorithm for 2000 steps**, which provides optimized geometry and standard placement of hydrogen atoms. While **explicit pH-dependent protonation adjustments** were not performed (as in Schrödinger's LigPrep), our approach ensures that ligands are in a reasonable and chemically consistent protonation state suitable for docking. This methodology has been widely adopted in many studies (Mahmud et al., 2021; Terefe and Ghosh, 2022; Joshi et al., 2025). We have revised the subsection "2.2. Preparation of ligands" with additional details and references.

Joshi, V., Bachhar, V., Mishra, S. S., Shukla, R. K., Kumar, N., & Duseja, M. (2025). Identification of potential anticancer phytochemicals of *Piper chaba* by comprehensive molecular docking, molecular dynamics simulation and in-vitro studies. *Chemistry & Biodiversity*, e202403269.

Mahmud, S., Mita, M. A., Biswas, S., Paul, G. K., Promi, M. M., Afrose, S., ... & Saleh, A. (2021). Molecular docking and dynamics study to explore phytochemical ligand molecules against the main protease of SARS-CoV-2 from extensive phytochemical datasets. *Expert Review of Clinical Pharmacology*, *14*(10), 1305-1315.

Terefe, E. M., & Ghosh, A. (2022). Molecular docking, validation, dynamics simulations, and pharmacokinetic prediction of phytochemicals isolated from *Croton dichogamus* against the HIV-1 reverse transcriptase. *Bioinformatics and Biology Insights*, *16*, 11779322221125605.

Comment 26: Full technique details regarding molecular docking computations should be considered in the revised manuscript, such as the grid spacing value.

Response 26. Thank you for the suggestion. In the revised manuscript, we have included the grid spacing used in the docking procedure (please refer to the revised subsection *2.4. Molecular docking*). The docking grid box was defined with a spacing of 0.375 Å, providing an appropriate resolution for sampling the ligand-receptor interactions within the active site. Other grid box parameters, including size and center coordinates, are also reported for reproducibility.

Comment 27: The reference of the utilized pdb code should be cited in the revised manuscript.

Response 27. We appreciate your comment. The reference (Istvan and Deisenhofer, 2001) for the protein structure (PDB ID: 1HWK) has now been properly cited in the revised section – *2.1. Protein Preparation*.

Istvan, E. S., & Deisenhofer, J. (2001). Structural mechanism for statin inhibition of HMG-CoA reductase. *Science*, *292*(5519), 1160-1164.

Comment 28: Did the authors perform equilibration for the investigated complexes prior to executing molecular dynamics simulations?

Response 28. Thank you very much for your critical observation. Yes, equilibration was thoroughly performed using Desmond's default multi-stage relaxation protocol before the 500 ns production run. These stages included restrained Brownian-dynamics NVT and NPT steps at 10 K, pocket solvation, restrained NPT equilibration, and unrestrained NPT equilibration prior to initiating the production simulation. The detailed equilibration process has been explicitly incorporated into subsection *2.8* ***Molecular dynamics (MD) simulation*** in the revised manuscript.

Reviewer 2

The manuscript titled "Computational Identification of Epifriedelanol and Derived Analogs from *Mikania cordata* as Potential HMG-CoA Reductase Inhibitors". In general, this is an interesting research topic approach, and a useful dataset has been provided. However, there are many important major points that must be addressed before considered for acceptance.

Comment 1. In Abstract, abbreviations that are used for the first time should be written in full name. (For Ex. HMG-CoA, ADMET, MM/GBSA….) Rewrite the Abstract.

Response 1. Thank you very much. We have rewritten the abstract with inclusion of full form where the abbreviations appear for the first time.

Comment 2. From which part of the *Mikania cordata* plant were the 92 phytocompounds extracted?

Response 2. We thank the reviewer for raising this point. We apologize for the earlier typographical error. The total number of phytocompounds extracted from *Mikania cordata* was 91, and when including the control drug Atorvastatin, the total number of ligands analyzed was 92. To clarify this, we have revised Table 1 and Table S1 and added an additional column specifying the plant part used for the extraction of each phytocompound.

Dr. M. Oliur Rahman
M.Sc. (Dhaka), Ph.D. (Japan), FLS (London)
Professor

Department of Botany
University of Dhaka
Dhaka-1000, Bangladesh
Phone: ++88 01717751718 (Cell); (02) 9661900 (off)
E-mail: oliur.bot@du.ac.bd

Comment 3. In Introduction part a short explanation on the traditional applications of Mikania cordata in cardiovascular / metabolic disorders would help bridge ethnomedicine with modern pharmacology.

Response 3. Thank you very much for your valuable suggestion. In the revised manuscript, we have included a brief explanation of the traditional applications of *Mikania cordata* relevant to cardiovascular and metabolic health with additional references. This addition helped to clearly bridge ethnomedicine with modern pharmacological investigation and strengthen the rationale for evaluating its phytoconstituents as potential HMG-CoA reductase inhibitors.

Comment 4. Cite recent references (Example: 10.1371/journal.pone.0325613, DOI:10.1371/journal.pone.0323702).

Response 4. We have cited the relevant recent references as recommended by the reviewer in the introduction part (inserted as reference number 11 and 12). Thank you.

Comment 5. A statement of the novelty of the manuscript has to be added at the end of the introduction.

Response 5. In the revised manuscript, we have added an explicit statement of novelty at the end of the introduction. The sentence now reads:

> "To the best of our knowledge, this study represents the first comprehensive computational assessment of *M. cordata* phytoconstituents for their potential to inhibit HMG-CoA reductase and modulate cholesterol metabolism."

Comment 6. Provide in separate Supplementary Table the structures of these 92 selected compounds.

Response 6. Thank you for your suggestion. Due to the large number of compounds and multiple associated parameters already presented in Supplementary Table S1 (e.g., IMPPAT ID/PubChem CID, molecular formula, plant part used, molecular weight, and binding affinity), inclusion of the 2D structures within the same table would have significantly affected its clarity and readability. Therefore, we have provided the two-dimensional chemical structures of all 91 phytocompounds and the control drug (Atorvastatin) separately in the supplementary Figure 2 (Fig S2) for better visualization and organization.

Comment 7. In Table 1, kindly mention the phytochemical names (From IMPHY014842 to IMPHY011562).

Response 7. In the revised manuscript, a new column has been added to Table 1 and Table S1 to include the names of all phytochemicals (from IMPHY014842 to IMPHY011562). As Reviewer 1 previously remarked that "*Table 1 is very long. It should be summarized, and other data transported into supporting information*," we have summarized Table 1 to present the top 24 compounds considering the binding affinity of over -6.0 kcal/mol, while retaining all entries in Supplementary Table S1 to provide a clear and complete overview of the entire phytochemical dataset rather than fragmented information.

Comment 8. Check the abbreviations throughout the manuscript (Ex. Line 221 The ADME, Table 3 ADME, Make it ADMET, ….. check carefully).

Response 8. We appreciate the reviewer's careful observation. In the original version, toxicity parameters were described only in the text, hence the use of "ADME." To ensure consistency and clarity, we have now incorporated the toxicity parameters into Tables 3 and 6 (revised as ADMET

Dr. M. Oliur Rahman
M.Sc. (Dhaka), Ph.D. (Japan), FLS (London)

Professor

Department of Botany
University of Dhaka
Dhaka-1000, Bangladesh
Phone: ++88 01717751718 (Cell); (02) 9661900 (off)
E-mail: oliur.bot@du.ac.bd

tables) and updated all instances of "ADME" to "ADMET" throughout the manuscript. All changes have been highlighted in yellow.

Comment 9. Docking/simulation software versions, parameters should be cited properly.

Response 9. Thank you for your suggestion. We have thoroughly revised the methodology sections of both molecular docking and molecular dynamics simulations to include complete software specifications, parameter settings, and relevant citations.

Comment 10. Clear and consistent figure is essential for readability and scientific accuracy.

Response 10. Thank you for your suggestion. We have revised all the previous figures and ensured consistency and readability. The figures in the PDF built by the submission system seems blurry. But the original figures are not blurry, we submitted 300 DPI high-resolution TIF files for all the figures following PACE (Preflight Analysis and Conversion Engine) as per guidelines of PLOS ONE and the supplementary figures as well. In the PDF built by the submission system, there is a hyperlink in the top right corner of the figure containing page, saying "Click here to access/download;Figure;Fig1.tif", please click on the link to download the high-resolution figure. As the figures have high-resolution and bigger size, the submission system might reduce the resolution in the PDF, to keep the PDF in smaller size.

Comment 11. Please provide good resolution figure with complete labelling. (For Ex. in Fig 9A label on X axis Time (nsec) font size is not same as Fig 9B). Please check for all figures X / Y-axis and maintain uniformity for all figures.

Response 11. Many thanks for your recommendation. We have improved all the figures thoroughly throughout the manuscript and supplementary files. The previous Figures 8, 9, 10, and 11 were combined together in the revised Figure 6 (Fig 6A,B for previous Fig 8; 6C,D for previous Fig 9; and 6E for previous Figs 10 and 11), as the reviewer suggested to reduce the number of figures. We have checked all the figures and ensured uniform labelling of X and Y axes in the revised figures.

Comment 12. Figures are many. Some figures could be moved to supplementary file.

Response 12. Thank you for your comment. In the revised manuscript, previous Figures 1, 4, 7, 12, and 13 have been moved to the supplementary section as Figures S1, S3, S4, S5, and S6, respectively. In response to the request of Reviewer 1 for additional validation analyses such as docking protocol validation, per-frame binding energy (MM/GBSA), hydrogen bond, and center-of-mass (COM) analyses, three new figures (Figures 1, 8, and 9) have been included. Consequently, the total number of figures has been reduced from previous 16 to present 11 in the revised manuscript.

Comment 13. There are some typing mistakes as well, and authors are advised to carefully proof-read the text.

Response 13. Thank you very much for your critical observation. The entire revised manuscript has been thoroughly proofread, and all typographical and grammatical errors have been corrected to ensure clarity and consistency throughout the text. The changes have been highlighted in yellow.

Comment 14. The computational results are promising, the manuscript would benefit from a clearer outline of future experimental plans. Suggestions for in vitro assays and in vivo models would enhance translational relevance.

Response 14. Thank you for your constructive feedback. In the revised manuscript, we have expanded the last paragraph of the discussion section to provide a clearer outline of future experimental plans,

Dr. M. Oliur Rahman
M.Sc. (Dhaka), Ph.D. (Japan), FLS (London)

Professor

Department of Botany
University of Dhaka
Dhaka-1000, Bangladesh
Phone: ++88 01717751718 (Cell); (02) 9661900 (off)
E-mail: oliur.bot@du.ac.bd

including specific *in vitro* assays (HMG-CoA reductase enzyme inhibition and cell-based cholesterol biosynthesis studies) and *in vivo* models (diet-induced hypercholesterolemic rats or mice) to enhance the translational relevance of the findings.

Comment 15. Modify conclusion part. A clearer link between results and practical implications would strengthen the conclusion.

Response 15. Thank you for your comment. The conclusion has been modified to establish a clearer link between the computational results and their practical implications. It now not only summarizes the key findings but also outlines potential experimental strategies, including *in vitro* enzyme inhibition assays, cell-based studies, and *in vivo* evaluation in hypercholesterolemic models, to confirm the translational relevance of the lead compounds.

Comment 16. Few references are not as per Journal format. The authors have to check the consistency of the reference format in the text as per the journal format.

Response 16. In the revised manuscript, we have carefully checked and corrected all references to ensure they fully comply with the journal's formatting requirements. All previously cited references have been updated, and additional references have been formatted consistently, strictly adhering to the journal guidelines. Thank you.

Reviewer 3

The manuscript titled "Computational Identification of Epifriedelanol and Derived Analogs from Mikania cordata as Potential HMG-CoA Reductase Inhibitors" presents an extensive in silico screening and molecular dynamics-based approach to identify plant-derived inhibitors of HMG-CoA reductase. While the study is relevant and methodologically ambitious, the current version of the manuscript is not suitable for publication due to incomplete methodological details, weak presentation of results, and several technical and formatting inconsistencies. The overall computational workflow is acceptable, but the lack of methodological validation, figure quality, and precise scientific justification limits the study's reproducibility and impact. The overall computational workflow is acceptable, but the lack of methodological validation, figure quality, and precise scientific justification limits the study's reproducibility and impact.

Major Issues

Comment 1. The molecular docking methodology lacks essential parameters such as grid box dimensions, exhaustiveness level, and scoring function.

Response 1. Thank you very much for your observation. The revised manuscript now provides all essential docking parameters: the grid box dimensions were set to 52 × 50 × 50 Å, with center coordinates at 8.416 (X), 3.586 (Y), and 16.056 (Z) Å. A grid spacing of 0.375 Å was applied to define the resolution of the docking search space. The exhaustiveness was set to 9 to ensure adequate sampling, and AutoDock Vina's scoring function (as EasyDock Vina was built based on AutoDock Vina), which combines empirical and knowledge-based potentials to estimate binding free energy in kcal/mol, was used to rank the docked poses (Trott and Olson, 2010). These revisions ensure complete transparency and reproducibility of the docking methodology.

Trott, O., & Olson, A. J. (2010). AutoDock Vina: Improving the speed and accuracy of docking with a new scoring function, efficient optimization, and multithreading. *Journal of Computational Chemistry*, *31*(2), 455-461.

Comment 2. The molecular dynamics (MD) section omits force field justification, charge assignment methods, and equilibration steps.

Response 2. Thank you very much for your critical observation. The revised manuscript (subsection 2.8) now includes a clear justification for using the OPLS4 force field, citing its improved torsional and non-bonded interaction accuracy and its prior successful application with the same target (1HWK) in a recent study (Chen et al., 2025). Additionally, the charge-assignment procedure in Maestro and the detailed multi-stage equilibration protocol have been fully described to ensure methodological completeness and reproducibility.

Chen, L., Han, D., Gu, C., & Huang, W. (2025). Biological effects of Calceolarioside A as a natural compound: Anti-ovarian cancer, anti-tyrosinase, and anti-HMG-CoA reductase potentials with molecular docking and dynamics simulation studies. *Molecular Biotechnology*, 1-13.

Comment 3. The authors should clearly state how atomic charges were computed for ligands and whether the system was equilibrated prior to production runs.

Response 3. Thank you. We have thoroughly revised the methodology part (subsection 2.8) of molecular dynamics simulation to clarify that ligand atomic charges were automatically assigned in Maestro during system preparation to ensure full OPLS4 compatibility. Moreover, a detailed description of Desmond's multi-stage equilibration process has been added, confirming that all systems were properly equilibrated before initiating the 500 ns production run.

Comment 4. The rationale for selecting the specific PDB entry (1HWK) must be clarified—newer, higher-resolution structures exist.

Response 4. We sincerely thank the reviewers for this insightful comment. The PDB structure **1HWK** (Istvan and Deisenhofer, 2001) was selected as it represents the first and most extensively validated X-ray crystal structure of human HMG-CoA reductase complexed with a statin inhibitor. With a resolution of 2.22 Å, 1HWK has served as a benchmark model in numerous molecular docking, inhibitor design, and molecular dynamics studies over the past two decades (Ali et al., 2025; Bano et al., 2025; Suhail et al., 2025). Its longstanding and consistent use in literature supports its structural reliability and reproducibility for computational analyses.

We are aware that more recent structures, such as **8PKN** (released January 2025; Cryo-EM, 2.26 Å) and **8S6B** (released February 2025; Cryo-EM, 2.06 Å), are available (Karuppasamy and van Rooyen, 2025). However, both of these models were determined by single-particle Cryo-EM, represent **shorter sequence coverage (423 aa)**, and correspond to different aggregation states (particle-based reconstructions). In contrast, 1HWK provides a complete 467-residue catalytic domain obtained via high-quality X-ray diffraction, with well-defined refinement statistics (R-Value Work = 0.212, R-Value Free = 0.235).

Therefore, despite the recent availability of Cryo-EM structures with comparable or slightly improved nominal resolution, 1HWK remains the most comprehensive, experimentally validated, and widely cited crystal structure of human HMG-CoA reductase-statin complex. This makes it the most appropriate and consistent choice for structure-based molecular docking and inhibitor interaction studies in the present work. The rationale has been discussed in the discussion section, highlighted in yellow.

Ali, S., Ali, U., & Jan, M. I. (2025). Hypolipidemic and HMG-CoA reductase inhibitory effects of 15-oxoursolic acid from *Rhododendron arboreum*: An In-vivo and In-silico Study. *Drug Development and Industrial Pharmacy*, 1-12.

Bano, S., Ansari, J. A., Ahsan, F., & Khan, A. R. (2025). Molecular docking, phytochemical screening and evaluation of *Balliospermum montanum* roots for its antihyperlipidemic potential. *Cell Biochemistry and Biophysics*, 1-18.

Istvan, E. S., & Deisenhofer, J. (2001). Structural mechanism for statin inhibition of HMG-CoA reductase. *Science*, *292*(5519), 1160-1164.

Karuppasamy, M., & van Rooyen, J. (2025). Cryo-EM structures of apo and atorvastatin-bound human 3-hydroxy-3-methylglutaryl-coenzyme A reductase. *Structural Biology and Crystallization Communications*, *81*(3), 118-122.

Suhail, P., Joy, J., Musliyarakath, N., Padmaraj, C. P., Theruvath, A., & Varghese, N. (2025). Bioactive compounds from *Carica papaya* (Papaya) fruit peel target key enzymes in diabetes and dyslipidemia: A molecular insight. *Biomedical and Biotechnology Research Journal*, *9*(3), 246-254.

Comment 5. Before docking experimental data, the performance of the docking setup should be evaluated (e.g., cross-docking or known inhibitor validation).

Response 5. Thank you very much for emphasizing the importance of docking validation prior to experimental ligand screening. To assess the reliability of our docking setup, we performed a known inhibitor-based validation using Atorvastatin, the reference ligand of HMG-CoA reductase. Since our study focused on the CASTp-predicted binding cavity, Atorvastatin was re-docked into this pocket using the same docking parameters applied for the phytochemical screening. The redocked pose was then aligned with the original pre-docking conformation (PubChem pose) using PyMOL, and the root-mean-square deviation (RMSD) was calculated. The redocked ligand reproduced the original geometry with an RMSD of 2.163 Å (please refer to the newly added Figure 1), confirming the reproducibility and reliability of the docking protocol for the CASTp-defined site. This validation demonstrates that our grid settings and docking parameters are suitable for accurate pose prediction of the screened ligands.

Comment 6. The post-MD analysis is incomplete. Include MM-GBSA/MM-PBSA binding free energy calculations over simulation trajectories, center-of-mass analysis, hydrogen bond persistence, and stability metrics.

Response 6. Thank you for your insightful suggestions. In the revised manuscript, we have incorporated per-frame MM-GBSA binding energy calculations, center-of-mass (COM) analysis, and hydrogen bond persistence metrics across the simulation trajectories. Additionally, ligand strain energy calculations have been included to provide a more comprehensive assessment of ligand-receptor stability. The corresponding results are presented in the newly added Figures 8 and 9 and Table 7, which have been cited appropriately in the main text.

Comment 7. Figures are of poor quality and lack clarity; non-informative or redundant visuals should be moved to the Supporting Information.

Response 7. Thank you for your suggestion. In the revised manuscript, the previous Figures 1, 4, 7, 12, and 13 have been transferred to the Supporting Information section as Figures S1, S3, S4, S5, and S6, respectively. Additionally, following the reviewer's recommendation for enhanced validation analyses including docking protocol validation, per-frame binding energy (MM/GBSA), hydrogen bond, and center-of-mass (COM) analyses, three new figures (Figures 1, 8, and 9) have been incorporated. As a result, the total number of figures has been streamlined from previous 16 to present 11 in the revised manuscript.

We have revised all the previous figures, improved figure legends, and ensured consistency and readability. The figures in the PDF built by the submission system seems blurry. But the original figures are not blurry, we have submitted 300 DPI high-resolution TIF files for all the figures following PACE (Preflight Analysis and Conversion Engine) as per guidelines of PLOS ONE and the supplementary figures as well. In the PDF built by the submission system, there is a hyperlink in the top right corner of the figure containing page, saying "Click here to access/download;Figure;Fig1.tif", please click on the link (as shown in the submission system-

Dr. M. Oliur Rahman
M.Sc. (Dhaka), Ph.D. (Japan), FLS (London)
Professor

Department of Botany
University of Dhaka
Dhaka-1000, Bangladesh
Phone: ++88 01717751718 (Cell); (02) 9661900 (off)
E-mail: oliur.bot@du.ac.bd

generated PDF) to download the high-resolution figure. As the figures have high-resolution and bigger size, the submission system might reduce the resolution in the PDF, to keep the PDF in smaller size.

Comment 8. Several tables are excessively long (e.g., Table 1) and should be summarized.

Response 8. Thank you for your suggestion. We have shortened Table 1 by retaining only the top 24 phytocompounds considering the binding affinely of over -6.0 kcal/mol, while the complete dataset comprising all 91 phytocompounds along with the control drug Atorvastatin has been moved to the supplementary section (Table S1). Additionally, following the recommendation of Reviewer 2, two new columns (Compound name and Plant part used) have been incorporated into both Table 1 and Table S1.

Comment 9. Numerous grammatical and typographical errors are present.

Response 9. Thank you for your observation. We have checked the grammar, corrected typographical errors and revised the full manuscript thoroughly. The changes have been highlighted in yellow.

Comment 10. Replace all hyphens ("-") with proper minus signs (–).

Response 10. We thank the reviewer for this valuable observation. All hyphens used to indicate negative numbers or mathematical operations have been replaced with the proper minus sign (−) throughout the manuscript. Hyphens that are part of standard compound terms (e.g., disability-adjusted, non-covalent) have been retained according to grammatical convention.

Comment 11. References are incomplete and inconsistent with PLOS ONE style.

Response 11. Thank you for pointing this out. All references have been thoroughly revised and reformatted following the guidelines of PLOS ONE reference style. Incomplete citations have been completed, and consistency in formatting has been ensured throughout the reference list.

Comment 12. Limit keywords to a maximum of five.

Response 12. We have reduced the number of keywords from previous six to present five.

Comment 13. The RMSF and RMSD analyses suggest some complexes are not stable. These results should be discussed critically instead of claiming high stability.

Response 13. We appreciate the reviewer's careful scrutiny of the Molecular Dynamics (MD) results and acknowledge that the global protein Cα RMSD (specifically the mean of 9.59 Å for EA2) could initially suggest system instability. We concur that reliance on Cα RMSD alone can be misleading, as it captures both local protein conformational changes and potential ligand dissociation. Additionally, we note that the Root Mean Square Fluctuation (RMSF) analysis of active-site residues shows slightly higher flexibility for EA2 and EA3 compared to epifriedelanol and the control, atorvastatin (average RMSF: Epifriedelanol = 1.54 Å, Atorvastatin = 1.35 Å; EA2 = 2.11 Å, EA3 = 2.17 Å). These elevated RMSF values primarily reflect local conformational adaptation of key residues (e.g., Pro813, Tyr533, Ile536) to accommodate the ligand's stereochemistry, while other critical residues (e.g., Ala556, Ala763) remain stable. Thus, the RMSF data complement the interpretation of global RMSD, representing normal dynamic adjustments rather than ligand dissociation or destabilization. To provide a robust assessment, we have integrated six orthogonal, ligand-centric, and structural compactness analyses over the 500 ns trajectories. These metrics support the conclusion that all ligands, including EA2, maintained stable, compact, and persistent binding within the active site, exhibiting **local conformational adaptation rather than**

dissociation. The stability of the protein-ligand complexes is strongly supported by the consistency across six independent analyses, demonstrating that the lead compounds often perform better than the control drug, atorvastatin, in key metrics of binding stability and compactness.

A. Ligand-Centric Positional Stability (Binding Retention)

Ligand-relative RMSD: All test compounds showed lower mean fluctuations compared to the control, Atorvastatin (6.55 ± 0.66 Å). Specifically, EA2 (4.05 ± 0.82 Å) demonstrated stable conformation and movement acceptable for a flexible ligand within the active site (Please refer to the **Figures S5 and S6** in the supplementary file).

Protein-Ligand Center-of-Mass (PL-COM) Distance: This metric directly refutes major displacement. Among all compounds, EA2 exhibited the lowest mean distance (1.63 ± 0.44 Å) with the least fluctuation. This suggests a highly consistent and close binding orientation throughout the 500 ns trajectory, contrasting with Atorvastatin, which showed the highest mean distance (2.05 ± 0.36 Å), indicating greater flexibility within the pocket (Please refer to the newly added Figure 8E).

B. Structural Compactness and Solvent Shielding

Radius of Gyration (Rg): All test complexes maintained consistent and stable structural compactness. The Rg values for the lead compounds (Epifriedelanol: 4.29 ± 0.03 Å; EA2: 4.21 ± 0.05 Å; and EA3: 4.18 ± 0.05 Å) were all lower than that of Atorvastatin (4.99 ± 0.08 Å), indicating a more compact overall structure upon binding (Please refer to the Figures S5 and S6).

Molecular Surface Area (MolSA): This metric remained steady with low standard deviations, directly confirming stable molecular surface exposure. The mean MolSA for EA2 (388.18 ± 2.19 $Å^2$) and the other lead compounds was considerably lower than Atorvastatin (490.85 ± 7.46 $Å^2$), suggesting the formation of more compact complexes with less exposed surface area (Please refer to the Figures S5 and S6).

Solvent Accessible Surface Area (SASA): Lower SASA values signify reduced solvent exposure, confirming the ligands are stably buried. The SASA for EA2 (124.38 ± 25.81 $Å^2$) was lower than the control (154.33 ± 24.26 $Å^2$), supporting a reduced solvent exposure and compact binding conformation (Please refer to the Figures S5 and S6).

C. Interaction Persistence

Hydrogen-Bond Cumulative Count: While Atorvastatin showed the highest cumulative count (1425), EA2 (896) and EA3 (940) demonstrated a significant and persistent number of polar contacts throughout the simulation, supporting continued engagement with the active site residues (Please refer to the **newly added Figure 8A–D**).

In summary, the increased global Cα RMSD for EA2, along with moderate local RMSF fluctuations, is interpreted as local conformational adaptation and protein flexibility, which is a normal aspect of dynamic binding. The consistency and low fluctuations observed across six independent, ligand-centric metrics (PL-COM distance, Ligand-relative RMSD, Rg, MolSA, SASA, and H-bonds) provide strong quantitative evidence that all the lead compounds maintained stable, compact, and persistently bound conformations throughout the simulation. We have revised the manuscript to reflect this detailed interpretation. The MD simulation section of the results part has been revised, highlighted in yellow with an additional figure (**Figure 8**). We have further discussed this observation in the discussion section, highlighted in yellow.

Dr. M. Oliur Rahman
M.Sc. (Dhaka), Ph.D. (Japan), FLS (London)
Professor

Department of Botany
University of Dhaka
Dhaka-1000, Bangladesh
Phone: ++88 01717751718 (Cell); (02) 9661900 (off)
E-mail: oliur.bot@du.ac.bd

Comment 14. The benefits and limitations of computing binding energy post-docking must be explained.

Response 14. We appreciate the reviewer's suggestion to discuss the benefits and limitations of computing binding energy post-docking. Post-docking MM/GBSA calculations provide rapid, quantitative estimates of binding affinity, allowing early-stage selection of promising compounds. In our study, this approach facilitated the identification of Epifriedelanol as the lead phytocompound and the selection of EA2 and EA3 as top analogs, with binding energies higher than the control drug Atorvastatin. However, MM/GBSA based on docking poses is limited because it relies on static conformations and does not account for protein and ligand flexibility, solvent dynamics, or time-dependent stability. To address these limitations, we performed 500 ns molecular dynamics simulations followed by MM/GBSA calculations across 1000 frames, providing a dynamic, time-averaged evaluation of binding free energies (Please refer to the newly added Figure 9 and Table 7). The results confirmed that Epifriedelanol, EA2, and EA3 maintained stronger and more stable interactions than Atorvastatin throughout the simulation. This workflow is consistent with previously reported studies: Shimu et al. (2022) applied MM/GBSA immediately after docking to shortlist phytochemical inhibitors prior to MD simulations; Ahmed et al. (2022) used a similar approach for natural compounds from *Piper betle* targeting alpha-amylase and alpha-glucosidase; and Ahmed et al. (2025) performed MM/GBSA over all frames of a 500 ns MD trajectory to robustly evaluate *Toxicodendron succedaneum* phytocompounds stability against ERK2, demonstrating that time-resolved post-MD binding energy calculations provide a more realistic and reliable assessment of ligand-protein interactions. Together, these examples underscore the effectiveness of combining post-docking and post-MD MM/GBSA calculations for confident lead compound identification. We have added the benefits and limitations of computing free binding energy after docking in the discussion section with additional references, highlighted in yellow.

Ahmed, S. S., Rahman, M. O., Hasan, M. S., Uddin, S. N., & Begum, M. (2025). In silico investigation of Toxicodendron succedaneum phytochemicals as extracellular signal-regulated kinase 2 inhibitors for non-small cell lung cancer therapy. *Medical Oncology*, *42*(7), 260.

Ahmed, S., Ali, M. C., Ruma, R. A., Mahmud, S., Paul, G. K., Saleh, M. A., ... & Islam, M. R. (2022). Molecular docking and dynamics simulation of natural compounds from betel leaves (Piper betle L.) for investigating the potential inhibition of alpha-amylase and alpha-glucosidase of type 2 diabetes. *Molecules*, *27*(14), 4526.

Shimu, M. S. S., Mahmud, S., Tallei, T. E., Sami, S. A., Adam, A. A., Acharjee, U. K., ... & Kim, B. (2022). Phytochemical compound screening to identify novel small molecules against dengue virus: a docking and dynamics study. *Molecules*, *27*(3), 653.

Comment 15. Clarify the difference between ADMET and "drug-likeness" — these are not synonymous.

Response 15. Thank you very much for your critical observation. We agree that ADMET and drug-likeness represent distinct aspects of compound evaluation. Accordingly, we have revised the methodology section to clarify this distinction. The updated text now reads:

"The pharmacokinetic and drug-likeness properties of the selected compounds were evaluated using the SwissADME server [45]. Subsequently, toxicity parameters were predicted separately using the STopTox server to assess potential adverse effects, completing the comprehensive ADMET evaluation [46]."

This revision ensures that the methodological description accurately reflects the separate evaluation of pharmacokinetic, drug-likeness, and toxicity properties.

Comment 16. Rewrite the conclusion to focus on the most significant results and implications for future in vitro validation. Avoid repetition of results.

Dr. M. Oliur Rahman
M.Sc. (Dhaka), Ph.D. (Japan), FLS (London)
Professor

Department of Botany
University of Dhaka
Dhaka-1000, Bangladesh
Phone: ++88 01717751718 (Cell); (02) 9661900 (off)
E-mail: oliur.bot@du.ac.bd

Response 16. Thank you very much for your suggestion. We have revised the conclusion to focus on the most significant results and to avoid repetition of the earlier sections. The new conclusion highlights the lead compounds' promising profiles, emphasizes their potential as natural cholesterol-lowering agents, and underscores the need for future *in vitro* and *in vivo* validation to substantiate their therapeutic efficacy.

Comment 17. Cite the reference for the PDB structure used.

Response 17. We appreciate your comment. The reference (Istvan and Deisenhofer, 2001) for the protein structure (PDB ID: 1HWK) has now been properly cited in the section – *2.1. Protein Preparation*.

Istvan, E. S., & Deisenhofer, J. (2001). Structural mechanism for statin inhibition of HMG-CoA reductase. *Science*, *292*(5519), 1160-1164.

Comment 18. Include details of ligand and receptor protonation states prior to docking.

Response 18. Thank you for highlighting the importance of ligand and receptor protonation states prior to docking.

For the receptor, the structure of human HMG-CoA reductase (PDB ID: 1HWK) was prepared using AutoDockTools v1.5.6, where polar hydrogens were added and Kollman partial charges were assigned. The protein was then energy-minimized using the GROMOS96 43B1 force field in SWISS-PDB Viewer to relieve steric clashes, optimize side-chain geometry, and obtain a low-energy conformation. While explicit pKa calculations were not performed, this approach follows widely adopted protocols in protein-ligand docking studies (Gonfa et al., 2023; Mahmud et al., 2021; Azmal et al., 2024).

For the ligands, all compounds were energy-minimized using OpenBabel v3.1.1.1 with the MMFF94 force field and the steepest descent algorithm for 2000 steps. This procedure ensures optimized geometry and standard placement of hydrogen atoms. Although pH-dependent protonation adjustments were not performed (e.g., as in Schrödinger's LigPrep), the resulting structures are chemically reasonable and suitable for docking, consistent with previous studies (Mahmud et al., 2021; Terefe and Ghosh, 2022; Joshi et al., 2025).

We have revised subsections "2.1. Protein preparation" and "2.2. Preparation of ligands" in the manuscript to include these details with additional references.

Azmal, M., Paul, J. K., Prima, F. S., Talukder, O. F., & Ghosh, A. (2024). An in silico molecular docking and simulation study to identify potential anticancer phytochemicals targeting the RAS signaling pathway. *PLoS One*, *19*(9), e0310637.

Gonfa, T., Temesgen, A., Erba, O., Mengesha, E. T., & Sivasubramanian, M. (2023). Phytochemicals analysis, in vitro antibacterial activities of extracts, and molecular docking studies of the isolated compounds from *Melhania zavattarii* Cufod leaves. *Journal of Tropical Medicine*, *2023*(1), 8820543.

Joshi, V., Bachhar, V., Mishra, S. S., Shukla, R. K., Kumar, N., & Duseja, M. (2025). Identification of potential anticancer phytochemicals of *Piper chaba* by comprehensive molecular docking, molecular dynamics simulation and in-vitro studies. *Chemistry & Biodiversity*, e202403269.

Mahmud, S., Mita, M. A., Biswas, S., Paul, G. K., Promi, M. M., Afrose, S., ... & Saleh, A. (2021). Molecular docking and dynamics study to explore phytochemical ligand molecules against the main protease of SARS-CoV-2 from extensive phytochemical datasets. *Expert Review of Clinical Pharmacology*, *14*(10), 1305-1315.

Terefe, E. M., & Ghosh, A. (2022). Molecular docking, validation, dynamics simulations, and pharmacokinetic prediction of phytochemicals isolated from *Croton dichogamus* against the HIV-1 reverse transcriptase. *Bioinformatics and Biology Insights*, *16*, 11779322221125605.

Dr. M. Oliur Rahman
M.Sc. (Dhaka), Ph.D. (Japan), FLS (London)
Professor

Department of Botany
University of Dhaka
Dhaka-1000, Bangladesh
Phone: ++88 01717751718 (Cell); (02) 9661900 (off)
E-mail: oliur.bot@du.ac.bd

Comment 19. Ensure consistency of docking and energy units to one decimal place.

Response 19. We have ensured consistency of docking and energy units to one decimal place throughout the manuscript and re-checked carefully. Thank you.

Comment 20. Improve figure legends for clarity and readability.

Response 20. Thank you for your suggestion. We have revised all the previous figures, improved figure legends, and ensured consistency and readability. The figures in the PDF built by the submission system seems blurry. But the original figures are not blurry, we have submitted 300 DPI high-resolution TIF files for all the figures following PACE (Preflight Analysis and Conversion Engine) as per guidelines of PLOS ONE and the supplementary figures as well. In the PDF built by the submission system, there is a hyperlink in the top right corner of the figure containing page, saying "Click here to access/download;Figure;Fig1.tif", please click on the link (as shown in the submission system-generated PDF) to download the high-resolution figure. As the figures have high-resolution and bigger size, the submission system might reduce the resolution in the PDF, to keep the PDF in smaller size.

Following these extensive revisions, guided by the insightful suggestions and comments of all reviewers, we have substantially improved the scientific rigor, clarity, and coherence of the manuscript. We believe that these comprehensive revisions have effectively addressed all reviewer concerns, and that the current version now meets the high standards of *PLOS ONE* and is suitable for publication in this esteemed journal.

Thank you very much for your time and cooperation.

Kind regards.

Dr. M. Oliur Rahman
Professor
Department of Botany
University of Dhaka
Dhaka 1000, Bangladesh
