# Supplementary material for: Computational identification of epifriedelanol and derived analogs from Mikania cordata as potential HMG-CoA reductase inhibitors
Source: PLoS One. 2026 Jan 6;21(1):e0340573. doi: 10.1371/journal.pone.0340573 (PMC12774364; doi:10.1371/journal.pone.0340573)
Supplement: Attachment — Submitted filename: Response_to_Reviewers_auresp_2.pdf [file pone.0340573.s012.pdf]

Dr. M. Oliur Rahman
M.Sc. (Dhaka), Ph.D. (Japan), FLS (London)

Professor

Department of Botany
University of Dhaka
Dhaka-1000, Bangladesh
Phone: ++88 01717751718 (Cell); (02) 9661900 (off)
E-mail: oliur.bot@du.ac.bd

Date: 22 December 22025

Manuscript Number: PONE-D-25-51503R1

Manuscript Title: "Computational Identification of Epifriedelanol and Derived Analogs from *Mikania cordata* as Potential HMG-CoA Reductase Inhibitors"

Dr. Chandrabose Selvaraj
Academic Editor
PLOS ONE

Subject: Response to Reviewers and Submission of Second-round revision for PONE-D-25-51503R1

Dear Dr. Selvaraj,

We sincerely thank you and the reviewers for the careful evaluation of our manuscript entitled *"Computational Identification of Epifriedelanol and Derived Analogs from Mikania cordata as Potential HMG-CoA Reductase Inhibitors"* (Manuscript ID: PONE-D-25-51503R1). We appreciate the opportunity to further revise our work and your continued consideration of the manuscript for publication in **PLOS ONE**.

The reviewers' insightful comments and constructive suggestions have been invaluable in improving the quality of the manuscript. We have carefully addressed each point raised and implemented revisions to enhance the scientific rigor, clarity, and overall coherence of the study. Below, we provide a detailed, point-by-point response to all editorial and reviewer comments.

RESPONSES TO THE COMMENTS OF THE EDITOR AND REVIEWERS

Editorial comments

Comment 1: If the reviewer comments include a recommendation to cite specific previously published works, please review and evaluate these publications to determine whether they are relevant and should be cited. There is no requirement to cite these works unless the editor has indicated otherwise.

Response 1: We thank the editor for this note. In the present review, the reviewer did not recommend citation of any specific previously published works. Therefore, no additional evaluation or citation was required.

Comment 2: Please review your reference list to ensure that it is complete and correct. If you have cited papers that have been retracted, please include the rationale for doing so in the manuscript text, or remove these references and replace them with relevant current references. Any changes to the reference list should be mentioned in the rebuttal letter that accompanies your revised manuscript. If you need to cite a retracted article, indicate the article's retracted status in the References list and also include a citation and full reference for the retraction notice.

Dr. M. Oliur Rahman
M.Sc. (Dhaka), Ph.D. (Japan), FLS (London)

Professor

Department of Botany
University of Dhaka
Dhaka-1000, Bangladesh
Phone: ++88 01717751718 (Cell); (02) 9661900 (off)
E-mail: oliur.bot@du.ac.bd

Response 2: We have thoroughly reviewed the entire reference list to ensure its accuracy, completeness, and compliance with PLOS ONE referencing guidelines. We confirm that no retracted articles were cited in the manuscript. During this revision, two additional references were added to support our response to Comment 1 of Reviewer 1, and these references have been formatted following PLOS ONE guidelines. The reference list and in-text citations were updated accordingly to ensure consistency and to avoid any anomalies. All changes to the references have been reflected in the revised manuscript.

Reviewer 1

In the revised version of the submitted manuscript entitled "Computational Identification of Epifriedelanol and Derived Analogs from Mikania cordata as Potential HMG-CoA Reductase Inhibitors", most of our comments were taken into account. Nevertheless, the comments listed below are still ambigious and must be solved. Accordingly, a minor revision is a must before approving the acceptance of the manuscript in the "PLOS ONE" journal.

Comment 1: As shown in Figure 1, the authors report an RMSD of 2.163 Å between the predicted docking pose and the experimental pose. However, it is well-established in the scientific literature that an RMSD value below 2.0 Å is generally required to validate a docking protocol as accurate and reliable. Therefore, the reported RMSD exceeds the commonly accepted threshold, raising concerns about the robustness of the docking validation presented in the manuscript.

Response 1: We thank the reviewer for raising the important point regarding the RMSD threshold used for docking validation. While an RMSD value of ≤ 2.0 Å is often cited as a benchmark for good pose reproduction, it is not a strict or universally enforced cutoff. Rather, RMSD values should be interpreted in the context of ligand flexibility, receptor rigidity, and conservation of key binding interactions.

Several well-cited studies have demonstrated that RMSD values slightly above 2.0 Å can still represent valid and reliable docking solutions. For example, Ramírez and Caballero (2018) categorized docking poses with RMSD values between **2.0 and 3.0 Å** as ***acceptable solutions*** that preserve the correct ligand orientation and key binding features. Similarly, Wu et al. (2020) reported a redocking RMSD of **2.1129** Å for the co-crystal ligand of human maltase-glucoamylase (PDB ID: 3TOP) and concluded that the docking strategy was valid, emphasizing that despite RMSD > 2.0 Å, the docked and crystallographic poses were well superimposed at hydrogen-bonding sites. In our study, the obtained RMSD of 2.163 Å falls within this accepted range and is comparable to values reported in the above-mentioned validated docking studies. We have clarified this point in the revised Discussion section and added the relevant references (highlighted yellow) to improve transparency and address the reviewer's concern.

Ramírez, D., & Caballero, J. (2018). Is it reliable to take the molecular docking top scoring position as the best solution without considering available structural data?. *Molecules*, *23*(5), 1038.

Wu, J., Hu, B., Sun, X., Wang, H., Huang, Y., Zhang, Y., ... & Yu, Z. (2020). In silico study reveals existing drugs as α-glucosidase inhibitors: Structure-based virtual screening validated by experimental investigation. *Journal of Molecular Structure*, *1218*, 128532.

Dr. M. Oliur Rahman
M.Sc. (Dhaka), Ph.D. (Japan), FLS (London)
Professor

Department of Botany
University of Dhaka
Dhaka-1000, Bangladesh
Phone: ++88 01717751718 (Cell); (02) 9661900 (off)
E-mail: oliur.bot@du.ac.bd

Comment 2: All abbreviations utilized in the abstract should be defined.

Response 2: Thank you. We have ensured that all abbreviations are clearly defined upon their first occurrence.

Comment 3: Full equations utilized for computing binding energy using the MM-GBSA approach over MD simulations should be mentioned in the revised manuscript.

Response 3: Thank you for your valuable suggestion. In response, we have now explicitly included the full equation for computing binding energy using the MM-GBSA approach over MD simulations at section "*2.9. Post-MD simulation MM/GBSA analysis*" in the revised manuscript. These additions are clearly indicated and highlighted in yellow for ease of identification.

Comment 4: 100 ps is not sufficient for the equilibration stage of the investigated complexes nowadays. It should be at least 10 ns.

Response 4: We thank the reviewer for this important comment regarding the equilibration duration. We would like to clarify that the 100 ps simulation mentioned in the manuscript refers specifically to the initial Brownian dynamics stage performed under NVT conditions at 10 K with restrained solute heavy atoms, which represents only the first step of a multi-stage equilibration protocol, rather than the entirety of the the equilibration process. Subsequent stages included short NVT and NPT simulations with restraints, followed by unrestrained NPT equilibration, ensuring gradual relaxation of solvent and protein-ligand interactions prior to the production phase.

Similar multi-step equilibration strategies with short initial simulations are widely used in contemporary molecular dynamics studies and are considered standard practice in the literature, including publications in **PLOS ONE**. For example, **Mohamed et al. (2024, PLOS ONE)** employed a 100 ps Brownian dynamics stage as the first step in a seven-stage equilibration protocol prior to a 200 ns production run. Similarly, **Naidoo et al. (2025)** and **Zhao et al. (2025)** applied initial 100 ps Brownian dynamics steps as part of their multi-stage equilibration protocols. These studies demonstrate that short initial equilibration steps, when combined with staged equilibration and followed by sufficiently long production simulations, are adequate to achieve thermodynamic stability.

Mohamed, G. A., Abdallah, H. M., Sindi, I. A., Ibrahim, S. R., & Alzain, A. A. (2024). Unveiling the potential of phytochemicals to inhibit nuclear receptor binding SET domain protein 2 for cancer: Pharmacophore screening, molecular docking, ADME properties, and molecular dynamics simulation investigations. ***PLoS ONE***, *19*(8), e0308913.

Naidoo, V., Achilonu, I., Mirza, S., Hull, R., Kandhavelu, J., Soobben, M., & Penny, C. (2025). Computational Modelling of Tunicamycin C Interaction with Potential Protein Targets: Perspectives from Inverse Docking with Molecular Dynamic Simulation. *Current Issues in Molecular Biology*, *47*(5), 339.

Zhao, R., Hou, L., Tesfagaber, W., Song, L., Zhang, Z., Li, F., ... & Zhao, D. (2025). Virtual Screening and Molecular Dynamics Simulation Targeting the ATP Domain of African Swine Fever Virus Type II DNA Topoisomerase. *Viruses*, *17*(5), 681.

Comment 5: What is the justification for a temperature of 300 K?

Response 5: Thank you for this pertinent question. The simulation temperature of **300 K** was chosen to approximate physiological conditions for **human HMG-CoA reductase** while

Dr. M. Oliur Rahman
M.Sc. (Dhaka), Ph.D. (Japan), FLS (London)

Professor

Department of Botany
University of Dhaka
Dhaka-1000, Bangladesh
Phone: ++88 01717751718 (Cell); (02) 9661900 (off)
E-mail: oliur.bot@du.ac.bd

maintaining computational stability. This temperature is widely adopted in the literature for MD simulations of HMG-CoA reductase-ligand complexes to mimic a biologically relevant environment and to ensure realistic atomic motions. For example, Mazumder et al. (2024, *Aspects of Molecular Medicine*), Khori et al. (2023, *Chemical Physics Impact*), and Son et al. (2013, *PLOS ONE*) all conducted MD simulations of HMG-CoA reductase complexes at 300 K, maintaining stable trajectories and reliable conformational sampling. These studies demonstrate that 300 K provides a physiologically relevant temperature suitable for capturing dynamic behavior, protein-ligand interactions, and thermodynamic stability over the course of long simulations.

Khori, V., Zahedi, M., Mirzaei, H., Jabbari, A., & Hosseini, S. G. (2023). Repurposing of monacolin K decorated BN nanoparticle on inhibition of **HMG-CoA reductase**: In silico approach. *Chemical Physics Impact*, *7*, 100384.

Mazumder, R., Choudhury, D., Sarkar, A., Ghosh, A., Debnath, S., Debnath, B., & Ghosh, R. (2024). In silico approach for identification of potential tetracyclic triterpenoids from mushroom as **HMG-CoA reductase** inhibitor. *Aspects of Molecular Medicine*, *4*, 100053.

Son, M., Baek, A., Sakkiah, S., Park, C., John, S., & Lee, K. W. (2013). Exploration of virtual candidates for human **HMG-CoA reductase** inhibitors using pharmacophore modeling and molecular dynamics simulations. ***PLoS ONE***, *8*(12), e83496.

Comment 6: Again, RMSF estimates the apo-receptor and ligand-soaked complex variance throughout the simulation time. However, the data are presented in Figure 6 that did not show the data of the apo-structure.

Response 6: Thank you for this valuable suggestion. In response, we have revised Figure 6 to include both RMSD and RMSF profiles of the apoprotein over the 500 ns MD simulation alongside the ligand-bound complexes. Specifically, the RMSD and RMSF profiles of the apoprotein are now shown in the revised Figs 6E and 6F, respectively. The Results section has been updated accordingly to reflect these additions.

Comment 7: Why did the authors perform the equilibration stage in the NVT conditions?

Response 7: We thank the reviewer for this insightful question. The initial **NVT equilibration** was employed to stabilize the system's temperature while restraining the protein and ligand heavy atoms. During this stage, the number of particles (N), system volume (V), and temperature (T) are kept constant, allowing the solvent molecules to relax around the solute without large volume fluctuations that could distort the protein-ligand complex.

After the temperature was stabilized under NVT conditions, we switched to **NPT equilibration**, allowing the system to adjust its pressure and density under a constant number of particles, pressure, and temperature. This stepwise NVT → NPT equilibration is a **widely accepted standard practice** in molecular dynamics simulations (e.g., Mohamed et al., 2024, PLOS ONE; Zhao et al., 2025, *Viruses*), ensuring gradual relaxation of both solvent and solute and preventing structural artifacts before the production run.

Mohamed, G. A., Abdallah, H. M., Sindi, I. A., Ibrahim, S. R., & Alzain, A. A. (2024). Unveiling the potential of phytochemicals to inhibit nuclear receptor binding SET domain protein 2 for cancer: Pharmacophore screening, molecular docking, ADME properties, and molecular dynamics simulation investigations. ***PLoS ONE***, *19*(8), e0308913.

Zhao, R., Hou, L., Tesfagaber, W., Song, L., Zhang, Z., Li, F., ... & Zhao, D. (2025). Virtual Screening and Molecular Dynamics Simulation Targeting the ATP Domain of African Swine Fever Virus Type II DNA Topoisomerase. *Viruses*, *17*(5), 681.

Comment 8: As illustrated in Figure 9, Atorvastatin shows positive binding energy values beyond 300 ns of MD simulation. These positive values imply a loss of binding affinity and indicate that Atorvastatin may no longer interact with the protein between 300 and 500 ns. Further explanation is required.

Response 8: We thank the reviewer for this insightful observation regarding the MM/GBSA binding free energy profile of atorvastatin. The transient appearance of positive ΔG_bind values for atorvastatin beyond ~300 ns does not necessarily indicate complete ligand dissociation. Instead, these values reflect increased conformational flexibility and a relative weakening of interaction strength compared to the lead compounds during the later stages of the simulation.

MM/GBSA binding free energy calculations are **highly sensitive to instantaneous conformational fluctuations**, solvent exposure, and changes in protein-ligand interaction geometry, particularly for flexible ligands such as atorvastatin. Consequently, short-lived positive ΔG_bind values may arise even when the ligand remains within the binding pocket, especially during periods of partial disengagement or increased mobility rather than full unbinding.

This interpretation is supported by ligand-centric stability analyses indicating that atorvastatin remained associated with the active site throughout the simulation. The **protein-ligand center-of-mass (PL-COM)** distance did not show any abrupt or sustained increase indicative of complete ligand dissociation, confirming continued spatial proximity to the catalytic pocket (**Fig 8E**). Additionally, hydrogen-bond analysis revealed that although the number of hydrogen bonds decreased during the later stages of the simulation, **intermittent interactions** persisted until the end of the simulation trajectory (**Fig 8D**). Together, these observations indicate that the positive MM/GBSA values observed after 300 ns reflect a weakening of binding interactions of atorvastatin rather than a complete loss of protein-ligand association. This clarification has been added to the Discussion section of the revised manuscript (highlighted in yellow).

Reviewer 2

The authors have responded well to the comments. The manuscript has improved in both scientific clarity and presentation. To further strengthen the manuscript, I suggest the following minor key points to be addressed to meet the journal’s formatting and quality expectations.

Comment 1: Please ensure consistency in figure labeling throughout the manuscript. For example, at line 523 it is written as 'Figs S5 and S6,' at line 564 as 'Fig 8A–D,' and elsewhere as 'Fig.8E.' Kindly maintain a uniform style for figure references across the text."

Response 1: Thank you for pointing out the inconsistency in figure labeling. We have carefully revised the manuscript to ensure a uniform style throughout. Specifically, the dot after “Fig.” has been removed, “Fig 8A–D” has been standardized to “Figs 8A–D,” and “Fig. 8E” has been corrected to “Fig 8E.” All figure references now follow a consistent formatting style across the entire manuscript.

Dr. M. Oliur Rahman
M.Sc. (Dhaka), Ph.D. (Japan), FLS (London)
Professor

Department of Botany
University of Dhaka
Dhaka-1000, Bangladesh
Phone: ++88 01717751718 (Cell); (02) 9661900 (off)
E-mail: oliur.bot@du.ac.bd

Comment 2: In results section line number 582, in the sentence 'epifriedelanol, EA2, EA3, and atorvastatin demonstrated mean free binding energies of −39.5, 583 −31.3, −43.7, and −21.4 kcal/mol, respectively,' Should be corrected as 'Epifriedelanol, EA2, EA3, and atorvastatin demonstrated mean free binding energies of −39.5, 583 −31.3, −43.7, and −21.4 kcal/mol, respectively. Please carefully check the entire MS for similar typographical or formatting errors to ensure consistency and clarity."

Response 2: Thank you very much for your critical observation. We have amended the anomaly and thoroughly revised the manuscript. All similar inconsistencies have been checked and corrected throughout the manuscript, highlighted in yellow.

Comment 3: Please avoid repeating the full species name 'Mikania cordata' throughout the MS. After the first mention, you may abbreviate it as M. cordata. (Ex. line no. 101, 288,340, etc).

Response 3: Thank you for the helpful suggestion. We have revised the manuscript to use the abbreviated form *M. cordata* after its first full mention. All subsequent occurrences throughout the text (including those at lines 101, 288, 340, and elsewhere) have been updated accordingly to maintain consistency and avoid repetition. All the changes have been highlighted in yellow.

Reviewer 3

Comment: In the revised manuscript entitled "Computational Identification of Epifriedelanol and Derived Analogs from Mikania cordata as Potential HMG-CoA Reductase Inhibitors", the authors considered all our comments. Consequently, the manuscript may be published in the Journal of PLOS ONE.

Response: Thank you very much for evaluating the revised manuscript. We sincerely appreciate your time, effort, and constructive comments, which significantly improved the quality and clarity of our work. We are pleased that the revised version meets the journal's standards, and we are grateful for your recommendation for publication.

Following these revisions, guided by the constructive comments and suggestions of the reviewers, the manuscript has been substantially improved in terms of scientific rigor, clarity, and coherence. We believe that all reviewer concerns have now been fully addressed and that the revised manuscript meets the editorial and scientific standards of **PLOS ONE**, making it suitable for publication.

Thank you very much for your time and consideration.

With kind regards.

Professor Dr. M. Oliur Rahman
Department of Botany
University of Dhaka
Dhaka 1000, Bangladesh
